# A Systematic Study of the Effect of Different Molecular Weights of Hyaluronic Acid on Mesenchymal Stromal Cell-Mediated Immunomodulation

**DOI:** 10.1371/journal.pone.0147868

**Published:** 2016-01-28

**Authors:** Alejandro Gómez-Aristizábal, Kyung-Phil Kim, Sowmya Viswanathan

**Affiliations:** 1 The Arthritis Program, Toronto Western Hospital, Toronto, ON, Canada; 2 Cell Therapy Program, Princess Margaret Cancer Centre, Toronto, ON, Canada; 3 Institute of Biomaterials and Biomedical Engineering, University of Toronto, Toronto, Ontario, Canada; Wake Forest Institute for Regenerative Medicine, UNITED STATES

## Abstract

**Introduction:**

Osteoarthritis (OA) is associated with chronic inflammation, and mesenchymal stromal cells (MSCs) have been shown to provide pain relief and reparative effects in clinical investigations. MSCs are often delivered with hyaluronic acid (HA), although the combined mechanism of action is not fully understood; we thus investigated the immunomodulatory effects of combining MSCs with different molecular weights (MW) of HA.

**Methods:**

HAs with MWs of 1.6 MDa (hHA), 150 kDa or 7.5 kDa, were added to MSCs alone or MSC-immune cell co-cultures. Gene expression analyses, flow cytometry and cytokine measurements were assessed to determine the effect of HAs on the MSC interactions with immune cells.

**Results:**

MSCs in the presence of HAs, in both normal and lymphocyte-conditioned medium, showed negligible changes in gene expression. While addition of hHA resulted in increased proliferation of activated lymphocytes, both in the presence and absence of MSCs, the overall combined effect was a more regulated, homeostatic one; this was supported by higher ratios of secreted IL10/IFNγ and IL10/IL2, in lymphocyte cultures, than with lower MW HAs or no HA, both in the presence and absence of MSCs. In addition, examination of monocyte-derived macrophages showed an increased M2 macrophage frequency (CD14+CD163+CD206+) in the presence of hHA, both with and without MSCs.

**Conclusions:**

hHA produces a less pro-inflammatory environment than lower MW HAs. Moreover, combining hHA with MSCs has an additive effect on the MSC-mediated immunomodulation, suggestive of a more potent combination treatment modality for OA.

## Introduction

Osteoarthritis (OA) is a progressive degenerative joint disorder, in which chronic inflammation plays an important role [1–3]. OA has the highest prevalence among arthritis types, with about 12% of the senior US population suffering from symptomatic knee OA [4]. Given the limited intrinsic healing capacity of cartilage, treatment options of osteoarthritis (OA) are typically limited to symptom alleviation rather than disease modification: including pain management, exercise and intra-articular hyaluronic acid (HA) injections [5].

HA therapy of OA can increase synovial fluid viscosity and may reduce pain [6,7]. However, the overall effect of HA (without considering MW, concentration or volume) based on comparisons with saline infusions, show small differences in ameliorating pain [7]. The relationship between MWs of HA and efficacy is inconclusive [7], although it appears that native high MW HAs (MW 800–1500 kDa) may provide better outcomes [8–11].

Given that the only definitive treatment for OA is prosthetic joint replacement with its attending morbidities [12], there is an unmet medical need to develop novel, disease-modifying therapies. One potential therapy is the use of mesenchymal stromal cells (MSCs), which is currently under extensive investigation, with 12 completed and 13 ongoing clinical trials [13–20]. In a number of animal models [21–26] the reparative effects of MSCs have also been demonstrated.

MSCs and HA have been used in combination in 5 out of 25 clinical trials where OA is treated with MSCs [13–20], but it is unclear whether this combination results in an improved therapeutic effect over HA or MSCs alone. Results from OA animal models treated with MSCs and HA combined are unclear: with evidence of additive, neutral or even negative effects [21,24,25].

There are no measurable effects of native, non- crosslinked, HA of different MWs in solution on MSC chondrogenesis [27]. Equally, little is known about how HAs may affect the immunomodulatory capacity of MSCs, likely an important therapeutic property of MSCs for OA [1]. In this paper, we systematically investigate for the first time the effect of different MWs of HA on the immunomodulatory capacity of MSCs. Different MWs of HA were tested, as our hypothesis was that high MW HA would be more anti-inflammatory than lower MW HAs, which increase the risk of OA progression [28]. The study objective was to determine how different MWs of HAs would affect MSC interactions with peripheral blood mononuclear cells (PBMCs), T helper (Th) cells and macrophages. The expression of selected MSC transcripts, involved in immunomodulation, trophic activity, angiogenesis, proliferation and chondrogenesis, was determined; the functional effects of HAs of different MWs were studied on the MSC-mediated inhibition of lymphocyte proliferation, focusing on Th cells due to their potential role in the progression of OA [1,3,29]. The effects of HAs on MSC interactions with monocyte-derived macrophages (MDMs) were also investigated given that macrophages are the most abundant immune cells in the OA synovium[1] and are involved in OA progression [2]. To simulate an inflammatory environment, interferon γ (IFNγ) was added, which affects MDMs [30,31], licenses MSCs [32] and is present in the OA synovial fluid [33]. For all experiments, MSCs and immune cells from healthy volunteers were used. There are no reported differences in MSC immunomodulation [34] or PBMCs between healthy donors and OA patients; and healthy samples provide a baseline that is comparable to published literature (i.e. control groups lacking HA can be compared to published data [32,35–37]).

## Materials and Methods

### Reagents

Native HAs of 1.6 MDa (hHA), 150 kDa and 7.5 kDa HA, derived from Streptococcus Pyogenes, were obtained from Lifecore Biomedical (Chaska, MN). HAs were used at 500 μg/ml in medium. IFNγ+ conditions contained 500 U/ml (25 ng/ml) of recombinant human IFNγ (US Biological, Salem, MA).

### Cell Isolation and Culture

The University Health Network Research Ethics Board approved the acquisition of human tissues (bone marrow and blood, both acquired from consenting donors at Princess Margaret Hospital): approval #s 06-446-CE and 14-7483-AE, respectively. All participants provided written informed consent to participate in this study.

MSCs were acquired as previously described [38] from fresh bone marrow drawn from the iliac crest of consenting donor volunteers, and expanded (up to passage 4–6) using MSC growth medium: low glucose DMEM supplemented with 10% fetal bovine serum (FBS, screened for MSC growth). MSCs were cryopreserved and thawed at least 3 days before the start of an experiment.

PBMCs were isolated by density gradient centrifugation on Ficoll-Paque (GE Healthcare Life Sciences, Mississauga, Canada) from fresh blood samples. Monocytes were isolated by positive magnetic selection of CD14+ cells (monocytes) from PBMCs, and CD4+ T (Th) cells were isolated by negative selection from the CD14 negative population (Milteny Biotec, San Diego, CA).

### Peripheral Blood Lymphocytes (PBL) and T Cell Proliferation Assays

PBMCs and Th cells were stained with carboxyfluorescein succinimidyl ester (Lifetechnologies, Burlington, ON) following the manufacturer’s recommendations. 96-well plates were seeded with 5, 10 or 20 x10^3^ MSCs in MSC growth medium one day before adding 1x10^5^ carboxyfluorescein succinimidyl ester-lymphocytes, with or without IFNγ or HAs, in complete medium: RPMI1640 with L-glutamine (80%), DMEM (10%), 1 mM sodium pyruvate, 1X Penicillin-Streptomycin and FBS (10%). Lymphocytes within PBMCs (PBLs) were activated with anti-CD3 (OKT3) and anti-CD28a (CD28.2) antibodies (eBioscience, San Diego, CA) at 0.2 μg/ml each. Dynabeads Human T-Activator CD3/CD28 (Lifetechnologies) were used to activate Th cells. After 4 and 5 days, for Th cells and PBMCs respectively, cells in suspension were harvested and mixed with propidium iodide, and read using a FC500 flow cytometer (Beckman Coulter, Mississauga, Ontario). Cell number was determined by algorithmic regression accounting for both proliferation and viability.

### T Regulatory Cell (Treg) Assay

156x10^3^ resting Th cells were added to 24-well plates with or without 31x10^3^ attached MSCs (5:1 ratio [37]). All cultures were performed with complete medium, with or without IFNγ or HAs. Cells were left in culture with no medium changes until flow cytometry characterization, on day 6 after seeding.

### MSC-Monocyte Coculture

100x10^3^ monocytes were added to 24-well plates with or without 31x10^3^ attached MSCs (~3:1 ratio). Complete medium supplemented with 10 ng/ml of human granulocyte-macrophage colony-stimulating factor (GM-CSF) (PeproTech, Rocky Hill, NJ), with or without IFNγ or HAs, was used. Four days later, both adherent and non-adherent cells were harvested for flow cytometry characterization. TrypLE (Lifetechnologies) was used for adherent cell detachment.

### Antibodies and Flow Cytometry

Cells were co-labeled with propidium iodide, to gate on viable populations. During data analysis, MSCs were excluded from MDMs by CD90 positive cell exclusion, and from Th cells by CD4 negative cell exclusion. Cells were characterized using a FC500 flow cytometer and analyzed on FlowJo (Treestar). Antibodies used were: FITC-CD4 (OKT4), PE-Cy7-CD14 (61D3), PE-CD206 (19.2), PE-CD127 (eBioRDR5) and PE-Cy7-CD25 (BC96) (eBioscience); FITC-CD163 (GHI/61) and APC-CD90 (5E10) (Biolegend, San Diego, CA).

### Cytokine Detection

Interleukin (IL)10, IL2, IL12, IFNγ (Biolegend) and IL1 receptor antagonist (IL1RA) (PeproTech) were detected using ELISA kits according to the manufacturers’ recommendations. Conditioned medium (CM) from activated Th cells and MDMs were harvested 4 days after seeding. Cells were removed from the CM by centrifugation at 1000xg for 10 min before storage at -80°C. Cytokine levels from activated Th cells were normalized to the number of viable cells on day 4 to account for the different proliferation rates; this normalization was not required for MDMs.

### Gene Expression

MSC samples were incubated with HAs for 1, 4 and 24 hours, or with PBMC-CM (with or without HAs for 4 hours), followed by RNA isolation and reverse transcription (RT) using the High Capacity cDNA RT Kit (Lifetechnologies). Quantitative RT-PCR was performed using the FastStart Universal SYBR Green Master (Roche, Indianapolis, IN) and primer nucleotide sequences (S1 Table). Relative expression levels were calculated using the 2-ΔΔCt method [39], with Beta-2-Microglobulin (B2M) as housekeeping gene.

PBMC-CM was produced by activating the PBLs in PBMCs for 5 days, as described above. Subsequently, PBMCs were washed and medium replaced with fresh complete medium and allowed to incubate for 24 hours to produce CM, that was filtered (0.45 μm) and stored at -80°C until use.

### Study Design and Statistical Analyses

**Fig 1:**
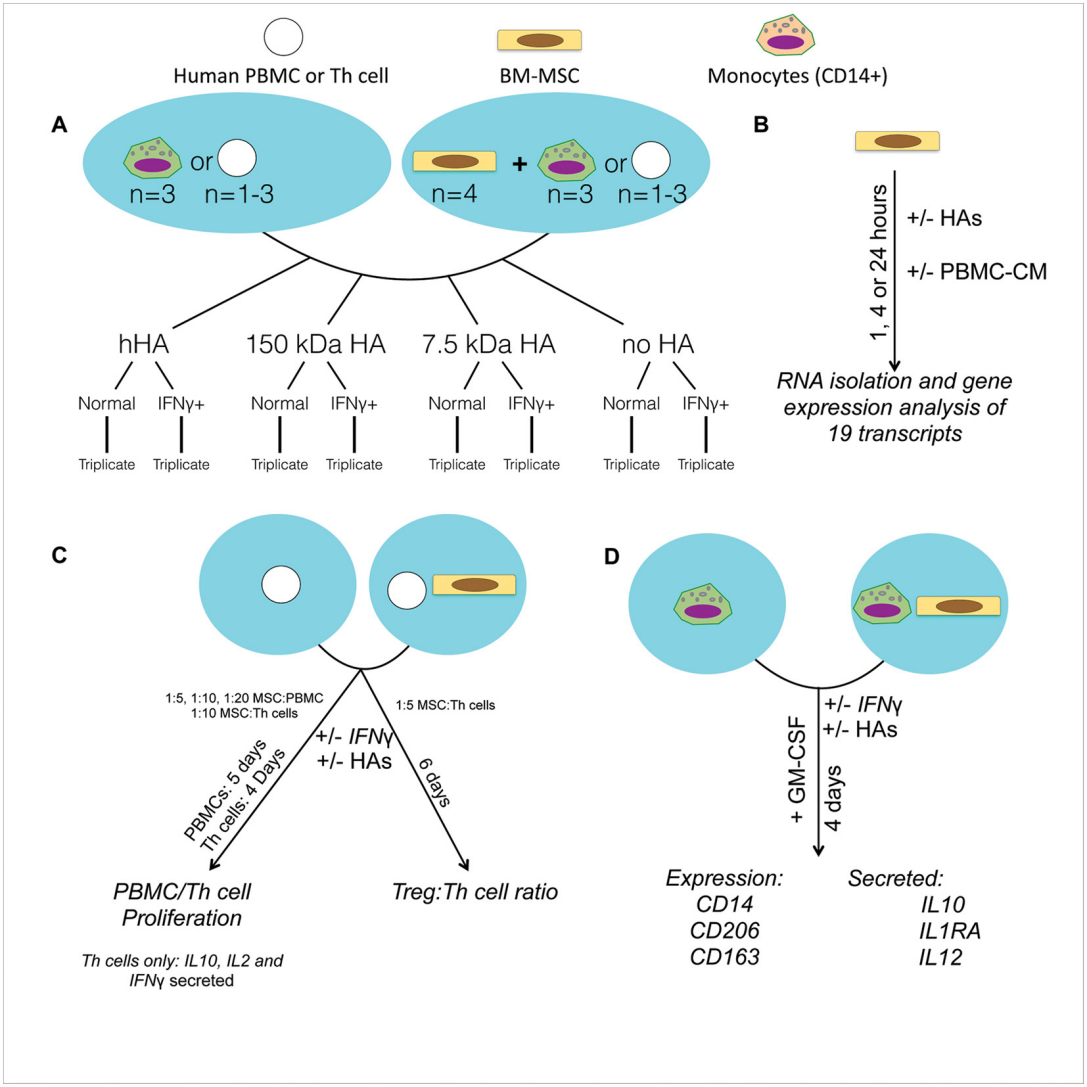
Overall experimental design. Legend of cell types shown on top. A) General experimental setup for MSC-immune cell interactions. A) Experimental setup to analyze the effects of HAs on the gene expression of MSCs. B) Experimental setup to analyze the effects of HAs on the capacity of MSCs to inhibit PBLs and Th cell proliferation, and to modulate Th cell activation (left); (right), the experimental setup to test the effects of HAs on the induction of Tregs by MSCs. C) Experimental setup to assay the effects of HAs on the immunomodulatory role of MSCs on MDMs.

MSCs were isolated from the bone marrow of 3–4 healthy donors. PBMCs were isolated from peripheral blood of 1 healthy independent donor unrelated to the MSC donors for the PBMC and Th cell proliferation assays; and from 3 independent donors for the Treg induction and monocyte experiments. The effect of 4 different MWs of HA (high, 150 kDa, 7.5 kDa, and no HA) was the main variable assessed in the presence of two conditions: normal and IFNγ+ (or PBMC-CM for gene expression analyses) (Fig 1). The effects of 4 MWs of HA under 2 different conditions on the MSC immunomodulatory capacity were analyzed in the following in vitro assays: 1) MSC gene expression analyses of 19 genes, PBL proliferation, Th cell proliferation and cytokine secretion, Treg induction, MDM phenotype switch and cytokine secretion.

Experiments were performed in triplicate or quadruplicate. As not much variability was seen in proliferation assays, data from a single PBMC or Th cell donor was used for analytical purposes; biological variation was accounted by using multiple MSC donors.

In Treg experiments, greater biological variability was observed in the Th cells vs. MSC donors, so data is also reported from 3 independent Th cell donors.

In the MDM experiments, due to limitations in monocyte number, three independent experiments, each with MSCs and monocytes from independent donors, were carried out to account for biological variation in both monocytes and MSC populations.

All data are expressed as mean of replicates ± 95% CI from a representative MSC donor; results from additional MSCs donors are shown in the supporting figures. However, statistical analyses were done on composite data from 3–4 MSCs donors, except for Tregs, where experiments were analyzed independently due to contrasting results between Th cell donors. Gene expression analyses were limited to a single MSC donor only when inflammatory conditions were used.

Statistical analyses were performed using generalized linear models with Gaussian distribution (S1 Equation) except for gene expression analyses. MSC donor sample variation was adjusted within the analytical model by accounting for it as a fixed factor (accounting MSC donor variability as a random factor using linear mixed-effect models showed no changes in the results). Measurements of the separate samples per donor were regarded as correlated observations. Treatments with 4 different HA values were considered fixed factors, and were assessed in the presence of two conditions: normal and IFNγ+, also considered as fixed factors. This allowed us to weigh donor-to-donor variability between MSCs from different donors and other variables to decipher the immune effector-MSC interaction effects in the presence of different MWs of HA. The validity of this analysis was evaluated by visually verifying that the residuals distribution had an approximate normal distribution and that there were no influencing outliers with a Cook’s value greater than 1. The qq plots of residuals were also verified. The analyses were valid in each case.

Post-hoc comparisons were done using the Tukey test with Holm-Bonferroni correction [Deducer [40] package for R. R Foundation, Vienna, Austria]. For gene expression the Student T-test was employed, and the effects of HA were taken as statistically independent observations. A log-transformation was applied to all data before analyses to approach normal distribution, in order to perform parametric tests, as non-parametric tests have little power with small sample sets. P-values below 0.05 were considered significant.

## Results

### HA Has a Negligible Effect on the Expression of Selected Genes

MSCs from 3 independent donors were analyzed for changes in the levels of 19 selected transcripts involved in HA signaling (CD44, TLR4, ICAM1, NFKBIA), immunomodulation (COX2, IDO, TSG6, TGFβ, HGF, PD-L1 and PD-L2), trophic activity (HGF, IL6), angiogenesis (VEGFA and CXCL8), proliferation (CYCLIND1), chondrogenesis (SOX9, ACAN, TGF-B), joint lubrication (PRG4) and catabolism (MMP3). No significant change in MSC gene expression upon exposure to HAs of different MWs was seen at any of the tested times: 1 hour, 4 hours and 24 hours (S2 Fig). To further explore whether licensing MSCs, by exposure to an inflammatory environment as in OA, would change the unresponsiveness to HAs, we exposed MSCs (single donor) to both PBMC-CM and HAs of different MWs and did not see any HA-mediated changes in gene expression although an effect of the inflammatory environment on MSCs was apparent (Table 1).

**Table 1:** Effect of HAs on the expression of selected MSC transcripts (fold change relative to no HA control).

Transcript	1.6 MDa HA	150 kDa HA	7.5kDa HA	no HA	1.6 MDa HA+PBMC-CM	150 kDa HA+PBMC-CM	7.5 kDa HA+PBMC-CM	no HA+PBMC-CM
**CD44**	1.2 ±0.2	1.1 ±0.2	1.0 ±0.2	1.0 ±0.1	0.9 ±0.1	0.9 ±0.1	0.8 ±0.1	0.8 ±0.1
**IL6**	1.0 ±0.1	1.0 ±0.1	1.0 ±0.3	1.0 ±0.3	2.1 ±0.2^††^	2.3 ±0.2^†††^	2.3 ±0.3^††^	1.6 ±0.9
**COX2**	0.7 ±0.1	0.6 ±0.1	0.6 ±0.2	1.0 ±0.4	0.7 ±0.1	0.9 ±0.1	0.7 ±0.1	0.8 ±0.1
**SOX9**	0.9 ±0.2	0.8 ±0.1	0.8 ±0.1	1.0 ±0.3	0.6 ±0.1	0.6 ±0.1	0.6 ±0.1	0.7 ±0.1
**PRG4**	0.9 ±0.2	0.9 ±0.1	0.8 ±0.3	1.0 ±0.3	0.6 ±0.1	0.7 ±0.1	0.6 ±0.2	0.7 ±0.3
**TLR4**	1.1 ±0.0	0.9 ±0.1	1.0 ±0.3	1.0 ±0.1	0.7 ±0.5	1.0 ±0.0	0.9 ±0.1	0.9 ±0.1
**ICAM1**	1.2 ±0.2	1.1 ±0.2	1.0 ±0.3	1.0 ±0.1	18.0 ±2.3^†††^	17.2 ±2.6^†††^	17.9 ±2.0^†††^	17.8 ±3.0^†††^
**CYCLIND1**	1.4 ±0.1	1.1 ±0.2	1.2 ±0.3	1.0 ±0.4	1.1 ±0.2	0.9 ±0.1	0.9 ±0.1	0.9 ±0.1
**NFKBIA**	1.3 ±0.4	1.3 ±0.5	1.2 ±0.3	1.0 ±0.1	1.5 ±0.2	1.4 ±0.2	1.4 ±0.3	1.4 ±0.2
**CXCL8**	0.4 ±0.1	0.5 ±0.0	0.5 ±0.2	1.0 ±0.7	5.0 ±0.5^†††^	5.1 ±1.0^†††^	5.1 ±0.7^†††^	4.9 ±1.0^†††^
**MMP3**	1.0 ±0.4	0.8 ±0.2	0.9 ±0.2	1.0 ±0.1	1.1 ±0.4	1.0 ±0.1	1.0 ±0.2	0.6 ±0.6
**ACAN**	1.1 ±0.1	1.3 ±0.6	1.0 ±0.1	1.0 ±0.1	0.8 ±0.2	0.8 ±0.1	0.8 ±0.1	0.8 ±0.1
**VEGFA**	0.8 ±0.1	0.8 ±0.1	0.8 ±0.1	1.0 ±0.2	0.6 ±0.1	0.5 ±0.1^††^	0.6 ±0.0^†^	0.7 ±0.1^†††^
**PDL1**	0.7 ±0.4	0.7 ±0.3	0.7 ±0.4	1.0 ±0.7	1.4 ±0.2	1.5 ±0.5	1.5 ±0.5	1.6 ±0.6
**PDL2**	1.1 ±0.2	1.2 ±0.5	1.1 ±0.2	1.0 ±0.2	1.5 ±0.3	1.4 ±0.1	1.4 ±0.2	1.3 ±0.1
**HGF**	1.2 ±0.3	0.9 ±0.1	1.0 ±0.2	1.0 ±0.2	1.0 ±0.1	0.9 ±0.2	0.9 ±0.2	0.9 ±0.2
**TGF-B**	1.2 ±0.2	1.0 ±0.1	1.0 ±0.1	1.0 ±0.1	0.8 ±0.0^††^	0.8 ±0.1	0.7 ±0.1^††^	0.9 ±0.2
**TSG-6**	1.0 ±0.1	1.0 ±0.1	1.1 ±0.2	1.0 ±0.2	0.7 ±0.0^††^	0.7 ±0.0^†^	0.8 ±0.1^††^	0.8 ±0.0
**IDO** ^a^	**-**	**-**	**-**	**-**	**+**^†††^	**+**^†††^	**+**^†††^	**+**^†††^

Expression is shown as mean± 95% CI of three experimental replicates from a representative MSC sample after 4 hours of exposure to HA. Relative transcript expression was also normalized to the expression of B2M. Highlights:

^†^<0.05;

^††^<0.01 and

^†††^ <0.001 relative to similar counterpart without PBMC-conditioned medium (CM).

^a^ Shown as + and - expression, no effects from HA when expressed.

### MSC-Mediated Inhibition of PBL Proliferation Is Antagonized by hHA

**Fig 2:**
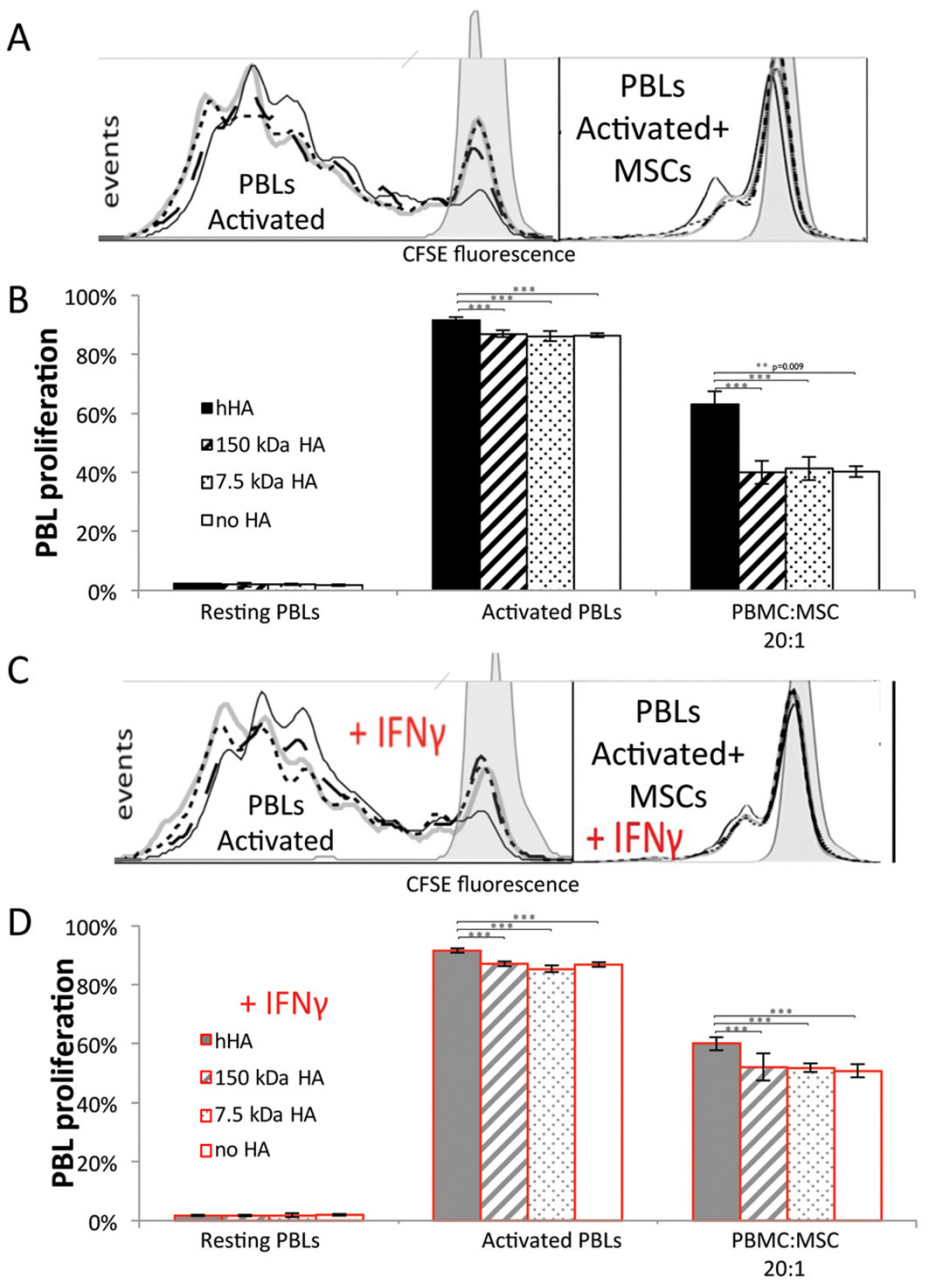
Effect of different MWs of HA on the MSC-mediated inhibition of PBL proliferation. (A) and (C) representative histograms of carboxyfluorescein succinimidyl ester (CFSE) fluorescence. Lines: Black solid line, hHA; black dashed line, 150 kDa HA; black dotted line, 7.5 kDa HA; and gray solid line, no HA. Gray shaded histogram represents resting PBMC control. (B) and (D) PBL proliferation from the whole PBMC population. (A) and (B), normal conditions. (C) and (D), IFNγ-supplemented conditions. Solid filled bars stand for hHA; hatched bars, 150 kDa HA; dotted bars, 7.5 kDa HA and empty bars, no HA. Black color, normal conditions and gray color with red frame, IFNγ-supplemented conditions. All data shown is from one representative MSC donor; additional data from three other donors and at different PBMC:MSC ratios are shown in S4 Fig. Error bars represent 95% CI. Statistical differences in the presence of MSCs account for MSCs from 4 donors, each tested in triplicate. Statistical differences in the absence of MSCs account for PBMCs from one donor tested in quadruplicate. * p<0.05, ** p<0.01 and ***<0.001.

hHA significantly increased the proliferation of activated PBLs (p<0.001 vs. lower MW HAs and no HA). MSCs significantly decreased activated PBL proliferation as previously shown [32], but combination with hHA resulted in higher PBL proliferation compared to no HA control (p = 0.009) (Fig 2). This effect was reproducible regardless of the presence of IFNγ (p<0.001) (Fig 2C and 2D), changes in MSC donors, alterations in the MSC:PBMC ratio (from 1:20 to 1:5)(S4 Fig) or the pre-stimulation of MSCs with IFNγ or agonists of TLR3 or 4 (S5 Fig). Digestion of hHA dampened this pro-mitogeneic effect on PBLs (S6 Fig).

### MSC-Mediated Inhibition of T Helper (Th) Cell Proliferation Is Antagonized by hHA

**Fig 3:**
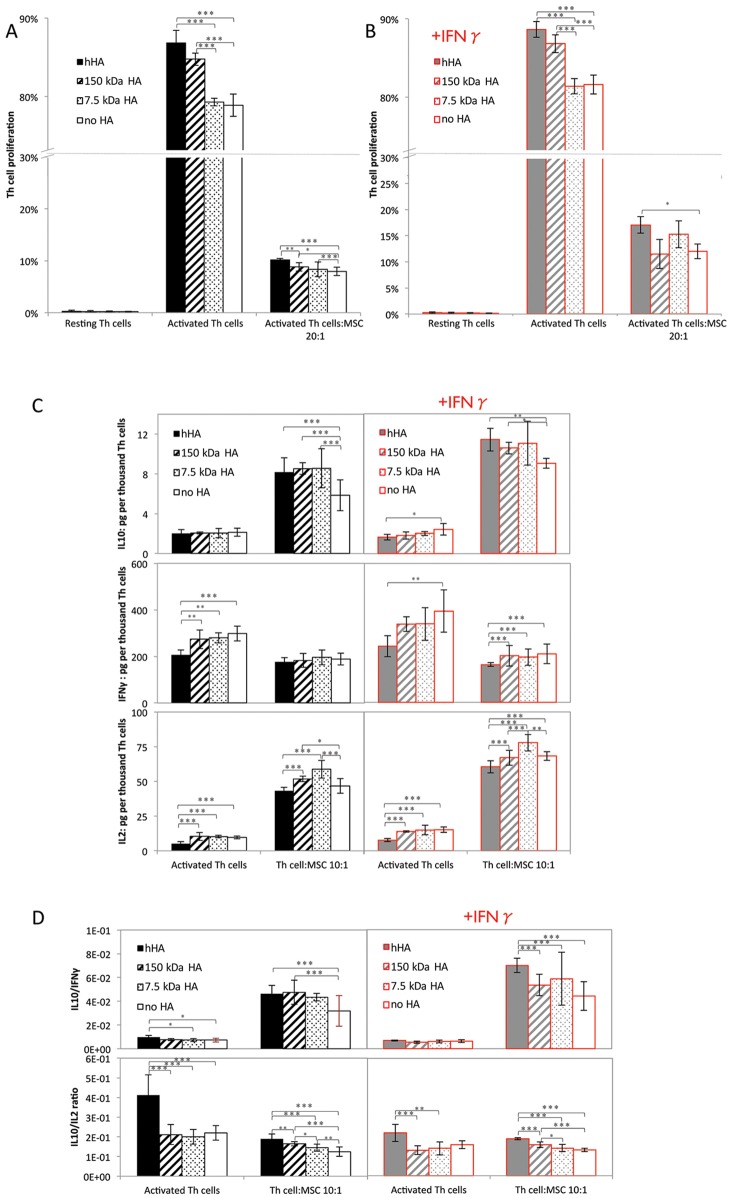
Effect of different MWs of HA on the interaction of MSCs and Th cells. Proliferation of Th cells, in the presence or absence of MSCs, without (A), and with IFNγ supplementation (B). (C) IL10, IFNγ and IL2 secreted levels in medium from activated Th cells with or without MSCs, and with or without IFNγ supplementation. (D) Ratios of secreted IL10/IFNγ, IL10/IL2. Solid filled bars stand for hHA; hatched bars, 150 kDa HA; dotted bars, 7.5 kDa HA and empty bars, no HA. Black color, normal conditions and gray color with red frame, IFNγ-supplemented conditions. All data shown is from one representative MSC donor; additional data from three other donors shown in S7 Fig. Statistical differences in the presence of MSCs account for MSCs from 4 donors, each tested in triplicate. Statistical differences in the absence of MSCs account for Th cells from one donor tested in quadruplicate. Error bars represent 95% CI. * p<0.05, ** p<0.01 and ***<0.001.

As with activated PBLs, hHA alone resulted in an increased proliferation of activated purified Th cells (p<0.001 vs. 7.5 kDa HA, and no HA conditions) (Fig 3A). Lower MW HA (150 kDa) also appeared to have a pro-mitogeneic effect, although to a lesser extent than hHA (p<0.001 vs. no HA condition). Combining hHA with MSCs resulted in an apparent decrease of MSC-mediated inhibition of activated Th cell proliferation (p<0.001 vs. no HA condition). Addition of IFNγ did not alter the pro-proliferative effect of hHA on MSC- Th cell interactions (Fig 3B).

### hHA Shifts Microenvironment to a More Regulatory One

To further understand the effect of combining hHA with MSCs, the Th cell secretory profile was analyzed by measuring levels of secreted IL10, IFNγ and IL2 to determine whether the microenvironment produced was more or less inflammatory (Fig 3C). The levels of these cytokines were negligible when measured in CM from MSCs alone or resting Th cells, indicating that activation of Th cells is required for the abundant secretion of these cytokines.

IL10 secretion, a known anti-inflammatory cytokine, was higher in the presence of MSCs than in their absence (p<0.001), even in the presence of IFNγ (Fig 3C top). Combining HAs of different MWs with MSCs increased the IL10 secretion even more, compared to the absence of HA (p<0.001 for all HAs vs. no HA condition). This HA effect on IL10 secretion was largely preserved even upon addition of IFNγ (p = 0.001 for hHA and p = 0.044 for 150 kDa HA vs. no HA condition), suggestive of an anti-inflammatory effect when HAs are combined with MSCs.

Levels of secreted IFNγ (Fig 3C middle), a pro-inflammatory cytokine, were lower upon addition of MSCs in all conditions compared to cultures without MSCs (normal, p = 0.007, and IFNγ+, p = 0.002). Adding hHA decreased the levels of secreted IFNγ in the absence of MSCs and in IFNγ-supplemented conditions with MSCs, suggestive of an anti-inflammatory role for hHA.

Secreted IL2 (Fig 3C bottom), a pro-mitogeneic inflammatory cytokine, was higher in all cultures with MSCs (p<0.001 vs. conditions without MSCs). Combining hHA with MSCs decreased IL2 secretion, compared to lower MW HAs, in all conditions tested (p<0.001 for hHA vs. 7.5kDa and 150 kDa HA conditions).

Overall, combining hHA with MSCs increased both the ratios of secreted IL10/IFNγ and IL10/IL2 (p<0.001 vs. no HA conditions) (Fig 3D), surrogate measures of regulatory vs. Th1 responses. The IL10/IFNγ ratio was higher in all cultures with MSCs (p<0.001 vs. without MSCs), confirming the immunomodulatory effect of MSCs. Importantly, adding hHA to MSCs increased this ratio in all conditions (p<0.001 vs. no HA conditions). Additionally, the IL10/IL2 ratio was increased by hHA, compared to lower MW HAs in all conditions tested (p<0.001 vs. 7.5 kDa and p = 0.003 vs. 150 kDa in normal conditions with MSCs; and p<0.001 vs. 7.5 kDa and 150 kDa HA in normal condition without MSCs; p<0.001 vs. 7.5 kDa HA and 150 kDa HA in IFNγ+ conditions with MSCs; p = 0.004 vs. 7.5 kDa HA and p<0.001 vs. 150 kDa HA in IFNγ+ conditions without MSCs).

### hHA Does Not Decrease T Regulatory Cells (Tregs) Frequency

**Fig 4:**
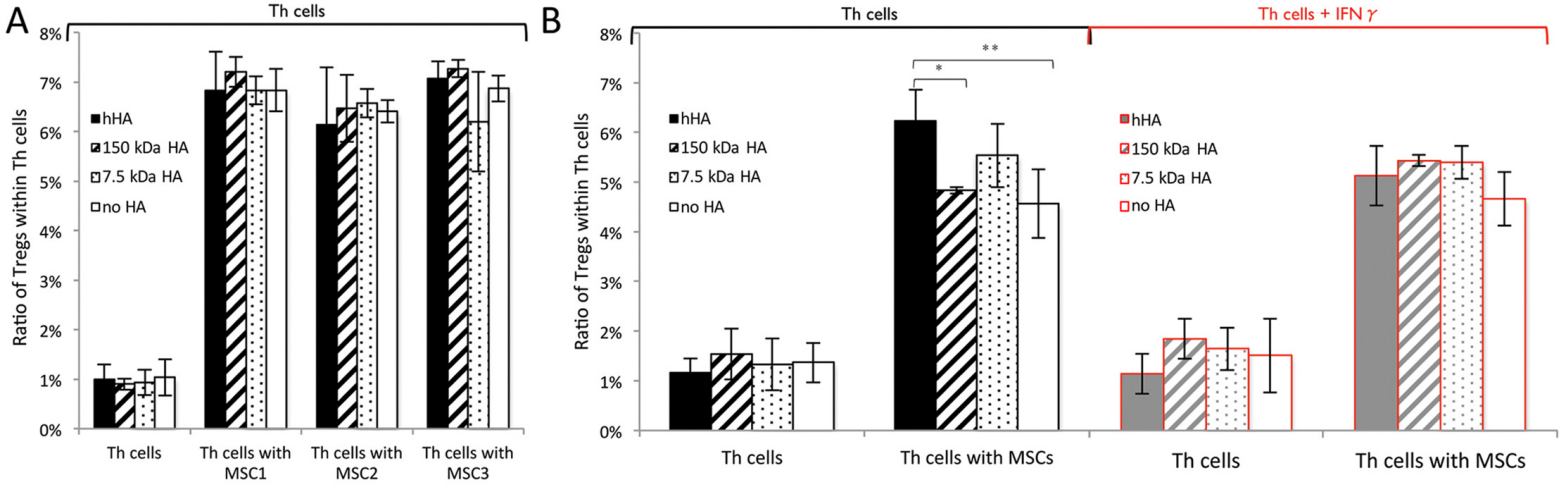
Effect of different MWs of HA on the MSC-mediated induction of Tregs. Frequency of Tregs (CD25+CD127-) within CD4+ T cells is shown (A, B). A) Effect of MSC donor-to-donor variability and different MWs of HA on Treg frequency. B) Effect of IFNγ-supplemented conditions on the Treg ratio from CD4+ T cells when MSCs and/or HAs are present. (A) and (B) show data from two separate experiments; normal conditions are shown in (B) for comparison purposes with IFNγ- supplemented conditions. Solid filled bars stand for hHA; hatched bars, 150 kDa HA; dotted bars, 7.5 kDa HA and empty bars, no HA. Black color, normal conditions and gray color with red frame, IFNγ-supplemented conditions. Each bar indicates the mean of triplicates replicates from MSCs or Th cell from one donor. Error bars represent 95% CI. * p<0.05, ** p<0.01 and ***<0.001.

Changes in the frequency of Tregs (CD4+CD25+CD127low [41]) were analyzed to further characterize hHA- and MSC-mediated effects on Th cells. MSCs were co-cultured with resting Th cells, thereby excluding other PBMC subpopulations. As expected, addition of MSCs led to an increased frequency of Tregs, with a reproducible effect between MSC samples from different donors (Fig 4A). Combining hHA with MSCs appeared to have a PBMC donor-specific effect: in one donor the combination increased the frequency of induced Tregs (p = 0.002 vs. no HA condition) (Fig 4B) and in another donor, it had no effect (Fig 4A, S8 Fig). Data on an effect of hHA (p = 0.089 vs. 150 kDa HA condition) on Treg frequency in the absence of MSCs is included for sake of completeness (S8 Fig). Conversely, lower MW HAs when combined with MSCs did not affect the frequency of Tregs (p = 0.164 and p = 1.00 for 7.5 kDa and 150 kDa HA, respectively, vs. no HA conditions) (Fig 4B). IFNγ had no effect on Treg frequency with or without HAs, although it dampened any potentiating effect of hHA when combined with MSCs (p = 0.565 for hHA vs. no HA conditions with IFNγ) (Fig 4B).

### Combining hHA with MSCs Potentiates the Induction of the M2 Monocyte-Derived Macrophage (MDM) Phenotype

**Fig 5:**
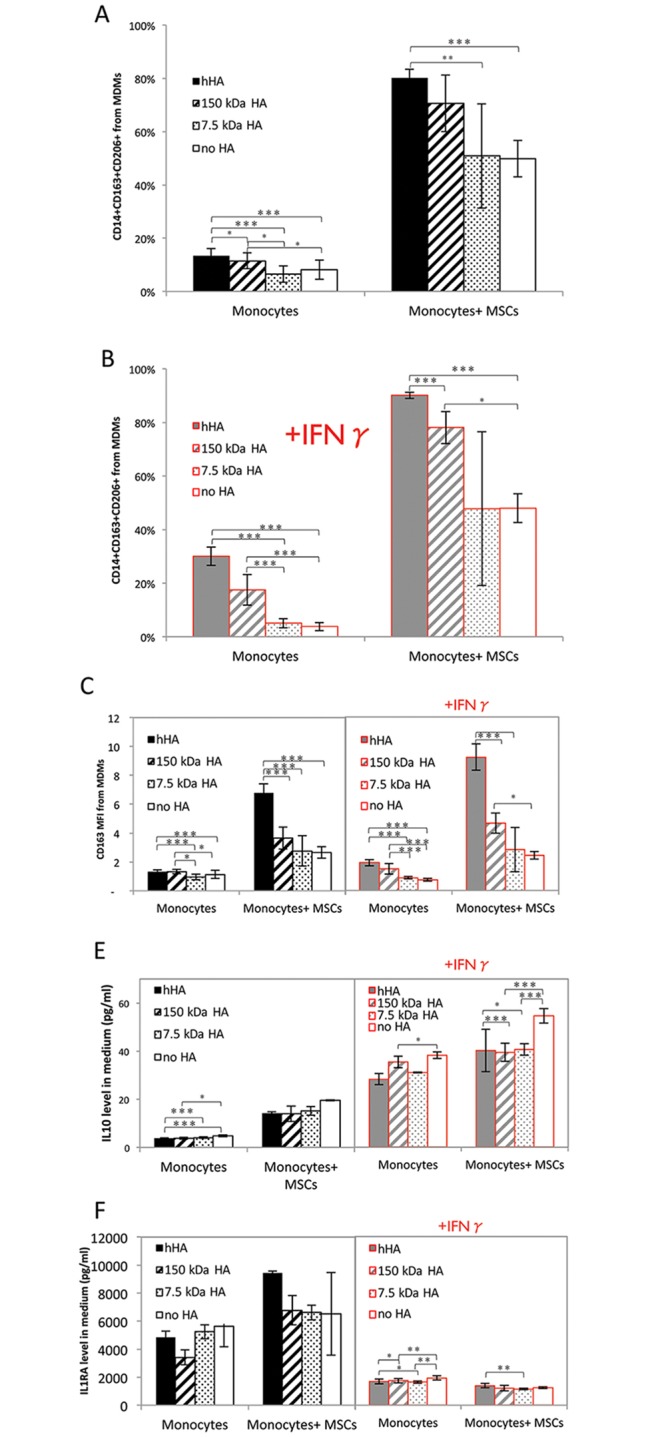
Effect of different MWs of HA on MSC-mediated induction of M2 MDMs. (A) and (B) Frequency of M2 MDMs within the whole MDM population. (C) mean fluorescence intensity (MFI) levels of CD163 from whole MDM population. All data shown is from one representative experiment; additional data from 3 MSC donors is shown in S9 Fig. (D) and (E) Levels of secreted IL10 and IL1RA as measured by ELISA. Solid filled bars stand for hHA; hatched bars, 150 kDa HA; dotted bars, 7.5 kDa HA and empty bars, no HA. Black color, normal conditions and gray color with red frame, IFNγ-supplemented conditions. Statistical differences account for data from three independent experiments; each with MSCs and monocytes from different donors in allogeneic co-cultures. Each bar indicates the mean of triplicates. Error bars represent 95% CI. * p<0.05, ** p<0.01 and ***<0.001.

Monocytes were induced to differentiate toward the macrophage phenotype with GM-CSF in the presence of combinations of different MWs of HAs with MSCs (Fig 1D). The surface phenotype of M2-MDMs was defined as CD14+CD206+CD163+ [35,36,42]. Under normal and IFNγ-supplemented conditions, adding MSCs increased the frequency of M2-MDMs compared to the absence of MSCs (p<0.001) (Fig 5A and 5B). Combining hHA with MSCs further increased this M2-MDMs frequency in both normal and IFNγ-supplemented conditions (p<0.001 vs. no HA conditions for both). Indeed, hHA alone increased the frequency of M2-MDMs in monocyte monocultures (p<0.001 vs. no HA conditions).

Cell surface antigen expression of CD163 (Fig 5C) was upregulated when MSCs were present compared to absence of MSCs (p<0.001). Adding hHA increased CD163 expression relative to no HA controls, especially in the presence of MSCs (p<0.001).

The microenvironment of MDMs was influenced by the presence of MSCs: levels of MDM-secreted IL10 were increased (p<0.001 vs. conditions without MSCs), confirming their immunomodulatory properties. Addition of IFNγ dampened this MSC-mediated increase (p<0.014 vs. conditions without MSCs). In contrast to the increase in the frequency of M2-MDMs, the combination of hHA with MSCs did not increase IL10 secretion (Fig 5D).

hHA also had no clear effect on IL1RA secretion. Low MW HAs inhibited the increase of IL1RA resulting from the addition of MSCs under normal conditions. IFNγ supplementation resulted in decreased MDM secreted IL1RA levels in the presence of MSCs (compared to conditions without MSCs, p<0.001) (Fig 5F). Secreted IL12 was not detected under any condition.

## Discussion

We systematically show for the first time that addition of native high MW of HA does not hinder, and in some measurements, improves the anti-inflammatory effects of MSCs. Fragments of HA, ~19 disaccharides for 7.5 kDa HA, do not activate the pro-inflammatory NF-κB pathway, as shown by the lack of up-regulation of NFKBIA [43] (Table 1, S2 Fig) compared to a positive control (LPS) (S3 Fig). This is unlike other reports of the effects of very low MW HA on chondrocytes [44,45] and thus suggests that MSCs can still exhibit anti-inflammatory behavior upon encountering HA fragments, such as in OA synovial fluid [28].

Addition of hHA increased lymphocyte proliferation. This is consistent with the co-stimulation of T cell proliferation upon CD44 crosslinking [46]. However, this contrasts with other reports [47] where lower proliferation was reported in the presence of high MW HA. This may be explained by a difference in the lymphocyte purification and activation approaches, and the possible presence of endotoxin in the HA preparations used [48].

While higher T cell proliferation is suggestive of a potential pro-inflammatory role of hHA, inconsistent with the historic use of HA (alone) for pain relief [6,7], a closer analysis showed that the activated Th cell microenvironment in the combined presence of hHA and MSCs was actually less inflammatory, with higher IL10/IFNγ and IL10/IL2 ratios (Fig 3D). In addition, Treg induction, while variable between lymphocyte donors, could be increased by the combination of hHA with MSCs (Fig 4B), as shown before in the absence of MSCs and under activating conditions [49]. While it is unknown whether Tregs could have a therapeutic effect in OA, they can induce M2-macrophages when activated [50] and decrease the Th1 response in OA [1,3,29].

hHA alone increased the frequency of M2-MDMs (Fig 5), and importantly had an additive effect when combined with MSCs. This immunoregulatory effect on macrophages is highly relevant to OA, as synovial macrophages are the most abundant immune cells in the OA synovium [1], and this suggests that combination therapies of MSC with hHA can modulate their role in OA progression. Macrophages may contribute to OA development by secreting metalloproteinases that destroy the cartilage matrix [2] and by inhibiting chondrogeneic differentiation of cartilage progenitors [31]. In our experiments, MDMs, while not exactly synovial macrophages, should mimic macrophages found in the OA environment, as they were induced to differentiate by exposure of monocytes to GM-CSF (instead of M-CSF) which is present in the OA synovium [51] and synovial fluid [33].

Levels of IFNγ in the OA joint tend to be approximately 200-fold lower [33] than our experimental conditions; relatively high levels were chosen based on previous evidence for both MSCs [32] and MDMs [30,31], and to assess the MSC- and HA-mediated effects under simplified pro-inflammatory conditions. While most experiments showed a consistent behavior between normal and IFNγ+ conditions, there were some differences on the effects mediated by MSCs: IFNγ decreased the levels of secreted IL1RA in MDM-MSC cocultures, and impaired the MSC-mediated enhancement of IL10 secretion. Previously, IFNγ has been shown to impair MDM-secretion of IL10 [30], but IFNγ also decreases IL1β secretion [52], suggesting that IFNγ decreases both anti- and pro-inflammatory cytokines and is not necessarily impairing the MSC mediated induction of the M2 phenotype.

All the MSCs transcripts analyzed remained unchanged when subjected to different MWs of HA, indicating that the mechanism of action of the HA effects is not mediated directly through MSCs. Additionally, the differential HA effects were unperturbed by TLR3/4-ligated MSCs (S5 Fig). We hypothesize that the HA effects observed are mediated by Th cells and MDMs; our data supports direct HA (alone) effects, particularly hHA, on both cell types. hHA due to its size, is able to induce CD44 clustering, an effect that can be inhibited by HA fragments [53], thus accounting for the differential effect of HAs of different MWs. This clustering allows interactions with adjacent receptors, i.e. CD44 and CXCR4 [54], and is associated with changes in the level of activation of the ERK1/2 pathway [53,54].

Our results support the suggestion that native high MW HAs (MW 800–1500 kDa) can provide better outcomes in the clinic [8–11]. In addition, high MW HAs have higher viscosities and are eliminated more slowly than lower MW HAs from the joints [55,56], providing increased biomechanical relief and prolonged interaction with joint components. High MW HAs are almost impermeable to most membrane barriers such as the skin [57], but the catabolic components within the joint eventually degrade HA allowing it to diffuse out. While crosslinking HA creates larger molecules which are more resistant to degradation [58], thus remaining longer within joints [56,59], our experiments were restricted to analyzing aqueous solutions of different MWs of native HA. HA crosslinking can alter its properties [60] and introduce confounding effects which would have prevented assessment of effects related to changes in MW of HA alone.

These investigations are limited to *in vitro* analyses to systematically examine the effects of combining human MSCs with HAs of different MWs on various immune subsets. It is thus possible to elucidate the direct effects of MSCs on lymphocytes and MDMs, while excluding other cell populations. Such analyses would not be possible in *in vivo* models where confounding immune rejection issues would be present when using human cells; and the use of animal cells may not be representative of the human immunological milieu [61].

Using a systematic evaluation, we have shown the immunomodulatory effects of different MWs of native HA in combination with MSCs on the function of lymphocytes and MDMs, both of which are prevalent in OA joints. While hHA had a pro-mitogeneic effect on PBLs and Th cells and dampened the anti-proliferative effect of MSCs, the overall effect including the effect on Tregs, macrophages and the secreted microenvironment was an anti-inflammatory one. Importantly, when combined with MSCs, hHA exhibits additive effects on inducing immune-effectors with a more regulatory profile, suggesting that a combination therapy may be more beneficial for the treatment of OA.

## Supporting Information

S1 Equation General linear model equation used for statistical analysis. (PDF)

S1 Fig HA MW estimation using agarose gel electrophoresis. (PDF)

S2 Fig Gene expression in MSCs after 1,4 and 24 hours of exposure to different HAs. (PDF)

S3 Fig Gene expression in MSCs after 1 hour exposure to 100 ng/ml LPS. (PDF)

S4 Fig Effect of different MWs of HA on the MSC-mediated inhibition of PBL proliferation. (PDF)

S5 Fig Effect of different MWs of HA on the MSC-mediated inhibition of PBL proliferation. (PDF)

S6 Fig Proliferation of PBLs in medium containing HAs or Hyaluronidase-digested HAs with or without MSCs. (PDF)

S7 Fig Effect of HAs on the interaction of MSCs and Th (CD4+ T cells) cells. (PDF)

S8 Fig Effect of HAs on the MSC mediated induction of Tregs. (PDF)

S9 Fig Effect of HAs on MSC-mediated induction of M2 MDMs. (PDF)

S1 Table Primer nucleotide sequences of the tested transcripts. (PDF)

S2 Table Abbreviations. (PDF)

## References

[1] de Lange-Brokaar BJE, Ioan-Facsinay A, van Osch GJVM, Zuurmond A-M, Schoones J, Toes REM, et al Synovial inflammation, immune cells and their cytokines in osteoarthritis: a review. Osteoarthritis Cartilage. 2012;20: 1484–1499. 10.1016/j.joca.2012.08.027 22960092

[2] Blom AB, van Lent PL, Libregts S, Holthuysen AE, van der Kraan PM, van Rooijen N, et al Crucial role of macrophages in matrix metalloproteinase–mediated cartilage destruction during experimental osteoarthritis: Involvement of matrix metalloproteinase 3. Arthritis Rheum. 2007;56: 147–157. 10.1002/art.22337 17195217

[3] Sakkas LI, Platsoucas CD. The role of T cells in the pathogenesis of osteoarthritis. Arthritis Rheum. 2007;56: 409–424. 10.1002/art.22369 17265476

[4] Lawrence RC, Felson DT, Helmick CG, Arnold LM, Choi H, Deyo RA, et al Estimates of the prevalence of arthritis and other rheumatic conditions in the United States. Part II. Arthritis Rheum. 2008;58: 26–35. 10.1002/art.23176 18163497 PMC3266664

[5] Ashford S, Williard J. Osteoarthritis: A review. Nurse Pract. 2014;39: 1–8. 10.1097/01.NPR.0000445886.71205.c4 24739424

[6] Vincent HK, Percival SS, Conrad BP, Seay AN, Montero C, Vincent KR. Hyaluronic Acid (HA) Viscosupplementation on Synovial Fluid Inflammation in Knee Osteoarthritis: A Pilot Study. Open Orthop J. 2013;7: 378–384. 10.2174/1874325001307010378 24093052 PMC3788189

[7] Colen S, van den Bekerom MPJ, Mulier M, Haverkamp D. Hyaluronic acid in the treatment of knee osteoarthritis: a systematic review and meta-analysis with emphasis on the efficacy of different products. BioDrugs Clin Immunother Biopharm Gene Ther. 2012;26: 257–268. 10.2165/11632580-000000000-00000 22734561

[8] Maheu E, Zaim M, Appelboom T, Jeka S, Trc T, Berenbaum F, et al Comparative efficacy and safety of two different molecular weight (MW) hyaluronans F60027 and Hylan G-F20 in symptomatic osteoarthritis of the knee (KOA). Results of a non inferiority, prospective, randomized, controlled trial. Clin Exp Rheumatol. 2011;29: 527–535. 21722501

[9] Karatay S, Kiziltunc A, Yildirim K, Karanfil RC, Senel K. Effects of Different Hyaluronic Acid Products on Synovial Fluid Levels of Intercellular Adhesion Molecule-1 and Vascular Cell Adhesion Molecule-1 in Knee Osteoarthritis. Ann Clin Lab Sci. 2004;34: 330–335. 15487709

[10] Petrella RJ, Decaria J, Petrella M. Long term efficacy and safety of a combined low and high molecular weight hyaluronic acid in the treatment of osteoarthritis of the knee. Rheumatol Rep. 2011;3 10.4081/rr.2011.e4

[11] Berenbaum F, Grifka J, Cazzaniga S, D’Amato M, Giacovelli G, Chevalier X, et al A randomised, double-blind, controlled trial comparing two intra-articular hyaluronic acid preparations differing by their molecular weight in symptomatic knee osteoarthritis. Ann Rheum Dis. 2012;71: 1454–1460. 10.1136/annrheumdis-2011-200972 22294639 PMC3414228

[12] Duchman KR, Gao Y, Pugely AJ, Martin CT, Callaghan JJ. Differences in short-term complications between unicompartmental and total knee arthroplasty: a propensity score matched analysis. J Bone Joint Surg Am. 2014;96: 1387–1394. 10.2106/JBJS.M.01048 25143499

[13] Viswanathan S, Gomez-Aristizabal A. Review of Patents and Commercial Opportunities Involving Mesenchymal Stromal Cells (MSCs) Therapies in Osteoarthritis. Recent Pat Regen Med. 2014;4: 1–15. 10.2174/2210296504666140307010938

[14] Wong KL, Lee KBL, Tai BC, Law P, Lee EH, Hui JHP. Injectable cultured bone marrow-derived mesenchymal stem cells in varus knees with cartilage defects undergoing high tibial osteotomy: a prospective, randomized controlled clinical trial with 2 years’ follow-up. Arthrosc J Arthrosc Relat Surg Off Publ Arthrosc Assoc N Am Int Arthrosc Assoc. 2013;29: 2020–2028. 10.1016/j.arthro.2013.09.074 24286801

[15] University of Jordan. Mesenchymal Stem Cells in Knee Cartilage Injuries. In: ClinicalTrials.gov [Internet]. 2014. Available: https://www.clinicaltrials.gov/ct2/show/NCT02118519.

[16] Chahal J, Viswanathan S. Human Autologous MSCs for the Treatment of Mid to Late Stage Knee OA. In: ClinicalTrials.gov [Internet]. 2015. Available: https://www.clinicaltrials.gov/ct2/show/NCT02351011.

[17] Espinoza F. A Study to Assess Safety and Efficacy of Umbilical Cord-derived Mesenchymal Stromal Cells in Knee Osteoarthritis. In: ClinicalTrials.gov [Internet]. 2015. Available: https://www.clinicaltrials.gov/ct2/show/NCT02580695.

[18] Shenzhen Hornetcorn Bio-technology Company, LTD, The Fifth Affiliated Hospital of Guangzhou Medical University. Human Umbilical Cord Mesenchymal Stem Cell Transplantation in Articular Cartilage Defect. In: ClinicalTrials.gov [Internet]. 2015. Available: https://www.clinicaltrials.gov/ct2/show/NCT02291926.

[19] Translational Biosciences. Clinical Study of Umbilical Cord Tissue Mesenchymal Stem Cells (UC-MSC) for Treatment of Osteoarthritis. In: ClinicalTrials.gov [Internet]. 2015. Available: https://www.clinicaltrials.gov/ct2/show/NCT02237846.

[20] Universidad de Navarra. Treatment of Osteoarthritis by Intra-articular Injection of Bone Marrow Mesenchymal Stem Cells With Platelet Rich Plasma (CMM-PRGF/ART). In: ClinicalTrials.gov [Internet]. 2015. Available: https://www.clinicaltrials.gov/ct2/show/NCT02365142.

[21] Murphy JM, Fink DJ, Hunziker EB, Barry FP. Stem cell therapy in a caprine model of osteoarthritis. Arthritis Rheum. 2003;48: 3464–3474. 10.1002/art.11365 14673997

[22] Singh A, Goel SC, Gupta KK, Kumar M, Arun GR, Patil H, et al The role of stem cells in osteoarthritis. Bone Jt Res. 2014;3: 32–37. 10.1302/2046-3758.32.2000187 PMC3926293 24526748

[23] Al Faqeh H, Nor Hamdan BMY, Chen HC, Aminuddin BS, Ruszymah BHI. The potential of intra-articular injection of chondrogenic-induced bone marrow stem cells to retard the progression of osteoarthritis in a sheep model. Exp Gerontol. 2012;47: 458–464. 10.1016/j.exger.2012.03.018 22759409

[24] Sato M, Uchida K, Nakajima H, Miyazaki T, Guerrero AR, Watanabe S, et al Direct transplantation of mesenchymal stem cells into the knee joints of Hartley strain guinea pigs with spontaneous osteoarthritis. Arthritis Res Ther. 2012;14: R31 10.1186/ar3735 22314040 PMC3392826

[25] Suhaeb AM, Naveen S, Mansor A, Kamarul T. Hyaluronic acid with or without bone marrow derived-mesenchymal stem cells improves osteoarthritic knee changes in rat model: a preliminary report. Indian J Exp Biol. 2012;50: 383–390. 22734248

[26] Mokbel AN, El Tookhy OS, Shamaa AA, Rashed LA, Sabry D, El Sayed AM. Homing and reparative effect of intra-articular injection of autologus mesenchymal stem cells in osteoarthritic animal model. BMC Musculoskelet Disord. 2011;12: 259 10.1186/1471-2474-12-259 22085445 PMC3232438

[27] Dvorakova J, Velebny V, Kubala L. Hyaluronan influence on the onset of chondrogenic differentiation of mesenchymal stem cells. Neuro Endocrinol Lett. 2008;29: 685–690. 18987597

[28] Band PA, Heeter J, Wisniewski H-G, Liublinska V, Pattanayak CW, Karia RJ, et al Hyaluronan molecular weight distribution is associated with the risk of knee osteoarthritis progression. Osteoarthritis Cartilage. 2015;23: 70–76. 10.1016/j.joca.2014.09.017 25266961 PMC4375131

[29] Shen P-C, Wu C-L, Jou I-M, Lee C-H, Juan H-Y, Lee P-J, et al T helper cells promote disease progression of osteoarthritis by inducing macrophage inflammatory protein-1γ. Osteoarthritis Cartilage. 2011;19: 728–736. 10.1016/j.joca.2011.02.014 21376128

[30] Verreck FAW, de Boer T, Langenberg DML, van der Zanden L, Ottenhoff THM. Phenotypic and functional profiling of human proinflammatory type-1 and anti-inflammatory type-2 macrophages in response to microbial antigens and IFN-γ- and CD40L-mediated costimulation. J Leukoc Biol. 2006;79: 285–293. 10.1189/jlb.0105015 16330536

[31] Fahy N, de Vries-van Melle ML, Lehmann J, Wei W, Grotenhuis N, Farrell E, et al Human osteoarthritic synovium impacts chondrogenic differentiation of mesenchymal stem cells via macrophage polarisation state. Osteoarthr Cartil OARS Osteoarthr Res Soc. 2014;22: 1167–1175. 10.1016/j.joca.2014.05.021 24911520

[32] Chinnadurai R, Copland IB, Patel SR, Galipeau J. IDO-Independent Suppression of T Cell Effector Function by IFN-γ–Licensed Human Mesenchymal Stromal Cells. J Immunol. 2014; 10.4049/jimmunol.1301828 24403533

[33] Vangsness CT Jr, Burke WS, Narvy SJ, MacPhee RD, Fedenko AN. Human knee synovial fluid cytokines correlated with grade of knee osteoarthritis—a pilot study. Bull NYU Hosp Jt Dis. 2011;69: 122–127. 22035391

[34] Hagmann S, Gotterbarm T, Müller T, Baesig A-M, Gantz S, Dreher T, et al The influence of bone marrow- and synovium-derived mesenchymal stromal cells from osteoarthritis patients on regulatory T cells in co-culture. Clin Exp Immunol. 2013;173: 454–462. 10.1111/cei.12122 23607395 PMC3949633

[35] Abumaree MH, Al Jumah MA, Kalionis B, Jawdat D, Al Khaldi A, Abomaray FM, et al Human placental mesenchymal stem cells (pMSCs) play a role as immune suppressive cells by shifting macrophage differentiation from inflammatory M1 to anti-inflammatory M2 macrophages. Stem Cell Rev. 2013;9: 620–641. 10.1007/s12015-013-9455-2 23812784

[36] Adutler-Lieber S, Ben-Mordechai T, Naftali-Shani N, Asher E, Loberman D, Raanani E, et al Human macrophage regulation via interaction with cardiac adipose tissue-derived mesenchymal stromal cells. J Cardiovasc Pharmacol Ther. 2013;18: 78–86. 10.1177/1074248412453875 22894882

[37] Del Papa B, Sportoletti P, Cecchini D, Rosati E, Balucani C, Baldoni S, et al Notch1 modulates mesenchymal stem cells mediated regulatory T-cell induction. Eur J Immunol. 2013;43: 182–187. 10.1002/eji.201242643 23161436

[38] Dayan V, Yannarelli G, Filomeno P, Keating A. Human mesenchymal stromal cells improve scar thickness without enhancing cardiac function in a chronic ischaemic heart failure model. Interact Cardiovasc Thorac Surg. 2012;14: 516–520. 10.1093/icvts/ivs048 22361124 PMC3329285

[39] Livak KJ, Schmittgen TD. Analysis of relative gene expression data using real-time quantitative PCR and the 2(-Delta Delta C(T)) Method. Methods San Diego Calif. 2001;25: 402–408. 10.1006/meth.2001.1262 11846609

[40] Fellows I. Deducer: A Data Analysis GUI for R. J Stat Softw. 2012;49: 1–15.

[41] Seddiki N. Expression of interleukin (IL)-2 and IL-7 receptors discriminates between human regulatory and activated T cells. J Exp Med. 2006;203: 1693–1700. 10.1084/jem.20060468 16818676 PMC2118333

[42] Mantovani A, Sica A, Sozzani S, Allavena P, Vecchi A, Locati M. The chemokine system in diverse forms of macrophage activation and polarization. Trends Immunol. 2004;25: 677–686. 10.1016/j.it.2004.09.015 15530839

[43] Bottero V, Imbert V, Frelin C, Formento J-L, Peyron J-F. Monitoring NF-kappa B transactivation potential via real-time PCR quantification of I kappa B-alpha gene expression. Mol Diagn J Devoted Underst Hum Dis Clin Appl Mol Biol. 2003;7: 187–194. 10.1007/BF03260037 15068390

[44] Campo GM, Avenoso A, Campo S, D’Ascola A, Traina P, Rugolo CA, et al Differential effect of molecular mass hyaluronan on lipopolysaccharide-induced damage in chondrocytes. Innate Immun. 2010;16: 48–63. 10.1177/1753425909340419 19710088

[45] Campo GM, Avenoso A, Campo S, D’Ascola A, Nastasi G, Calatroni A. Small hyaluronan oligosaccharides induce inflammation by engaging both toll-like-4 and CD44 receptors in human chondrocytes. Biochem Pharmacol. 2010;80: 480–490. 10.1016/j.bcp.2010.04.024 20435021

[46] Föger N, Marhaba R, Zöller M. CD44 supports T cell proliferation and apoptosis by apposition of protein kinases. Eur J Immunol. 2000;30: 2888–2899. 10.1002/1521-4141(200010)30:10<2888::AID-IMMU2888>3.0.CO;2-4 11069071

[47] Darżynkiewicz Z, Balazs EA. Effect of connective tissue intercellular matrix on lymphocyte stimulation: I. Suppression of Lymphocyte stimulation by hyaluronic acid. Exp Cell Res. 1971;66: 113–123. 10.1016/S0014-4827(71)80018-6 5104191

[48] Shiedlin A, Bigelow R, Christopher W, Arbabi S, Yang L, Maier RV, et al Evaluation of Hyaluronan from Different Sources: Streptococcus zooepidemicus, Rooster Comb, Bovine Vitreous, and Human Umbilical Cord. Biomacromolecules. 2004;5: 2122–2127. 10.1021/bm0498427 15530025

[49] Bollyky PL, Falk BA, Long A, Preisinger A, Braun KR, Wu RP, et al CD44 co-stimulation promotes FoxP3+ regulatory T-cell persistence and function via production of IL-2, IL-10 and TGF-beta. J Immunol Baltim Md 1950. 2009;183: 2232–2241. 10.4049/jimmunol.0900191 PMC3057032 19635906

[50] Tiemessen MM, Jagger AL, Evans HG, van Herwijnen MJC, John S, Taams LS. CD4+CD25+Foxp3+ regulatory T cells induce alternative activation of human monocytes/macrophages. Proc Natl Acad Sci. 2007;104: 19446–19451. 10.1073/pnas.0706832104 18042719 PMC2148309

[51] Farahat MN, Yanni G, Poston R, Panayi GS. Cytokine expression in synovial membranes of patients with rheumatoid arthritis and osteoarthritis. Ann Rheum Dis. 1993;52: 870–875. 10.1136/ard.52.12.870 8311538 PMC1005218

[52] Baeva LF, Lyle DB, Rios M, Langone JJ, Lightfoote MM. Different molecular weight hyaluronic acid effects on human macrophage interleukin 1β production. J Biomed Mater Res A. 2013; 10.1002/jbm.a.34704 23533059

[53] Yang C, Cao M, Liu H, He Y, Xu J, Du Y, et al The High and Low Molecular Weight Forms of Hyaluronan Have Distinct Effects on CD44 Clustering. J Biol Chem. 2012;287: 43094–43107. 10.1074/jbc.M112.349209 23118219 PMC3522304

[54] Fuchs K, Hippe A, Schmaus A, Homey B, Sleeman JP, Orian-Rousseau V. Opposing effects of high- and low-molecular weight hyaluronan on CXCL12-induced CXCR4 signaling depend on CD44. Cell Death Dis. 2013;4: e819 10.1038/cddis.2013.364 24091662 PMC3824673

[55] Brown T, Laurent U, Fraser. Turnover of hyaluronan in synovial joints: elimination of labelled hyaluronan from the knee joint of the rabbit. Exp Physiol. 1991;76: 125–134. 10.1113/expphysiol.1991.sp003474 2015069

[56] Larsen NE, Dursema HD, Pollak CT, Skrabut EM. Clearance kinetics of a hylan-based viscosupplement after intra-articular and intravenous administration in animal models. J Biomed Mater Res B Appl Biomater. 2012;100B: 457–462. 10.1002/jbm.b.31971 22102374

[57] Essendoubi M, Gobinet C, Reynaud R, Angiboust JF, Manfait M, Piot O. Human skin penetration of hyaluronic acid of different molecular weights as probed by Raman spectroscopy. Skin Res Technol Off J Int Soc Bioeng Skin ISBS Int Soc Digit Imaging Skin ISDIS Int Soc Skin Imaging ISSI. 2015; 10.1111/srt.12228 25877232

[58] Sall I, Férard G. Comparison of the sensitivity of 11 crosslinked hyaluronic acid gels to bovine testis hyaluronidase. Polym Degrad Stab. 2007;92: 915–919. 10.1016/j.polymdegradstab.2006.11.020

[59] Edsman K, Hjelm R, Lärkner H, Nord LI, Karlsson A, Wiebensjö Å, et al Intra-articular Duration of DurolaneTM after Single Injection into the Rabbit Knee. Cartilage. 2011;2: 384–388. 10.1177/1947603511400184 26069596 PMC4297137

[60] Ottaviani RA, Wooley P, Song Z, Markel DC. Inflammatory and Immunological Responses to Hyaluronan Preparations. J Bone Jt Surg Am. 2007;89: 148–157. 10.2106/JBJS.E.01135 17200322

[61] Seok J, Warren HS, Cuenca AG, Mindrinos MN, Baker HV, Xu W, et al Genomic responses in mouse models poorly mimic human inflammatory diseases. Proc Natl Acad Sci. 2013;110: 3507–3512. 10.1073/pnas.1222878110 23401516 PMC3587220

