# Supplementary material for: A Systematic Study of the Effect of Different Molecular Weights of Hyaluronic Acid on Mesenchymal Stromal Cell-Mediated Immunomodulation
Source: PLoS One. 2016 Jan 28;11(1):e0147868. doi: 10.1371/journal.pone.0147868 (PMC4731468; doi:10.1371/journal.pone.0147868)
Supplement: S1 Equation — (PDF) [file pone.0147868.s001.pdf]

$$\log(Measurement) = \beta 0 + Donor \cdot (MSC\!: responder\ ratio) \cdot \beta 1 + HAtype \cdot \beta 2 + (IFNsupplementation) \cdot \beta 3$$

Equation 1: General linear model equation used for statistical analysis. βx, parameters to be estimated. Variables within parenthesis were included where needed.
