# Supplementary figures and images for: A Systematic Study of the Effect of Different Molecular Weights of Hyaluronic Acid on Mesenchymal Stromal Cell-Mediated Immunomodulation
Source: PLoS One. 2016 Jan 28;11(1):e0147868. doi: 10.1371/journal.pone.0147868 (PMC4731468; doi:10.1371/journal.pone.0147868)

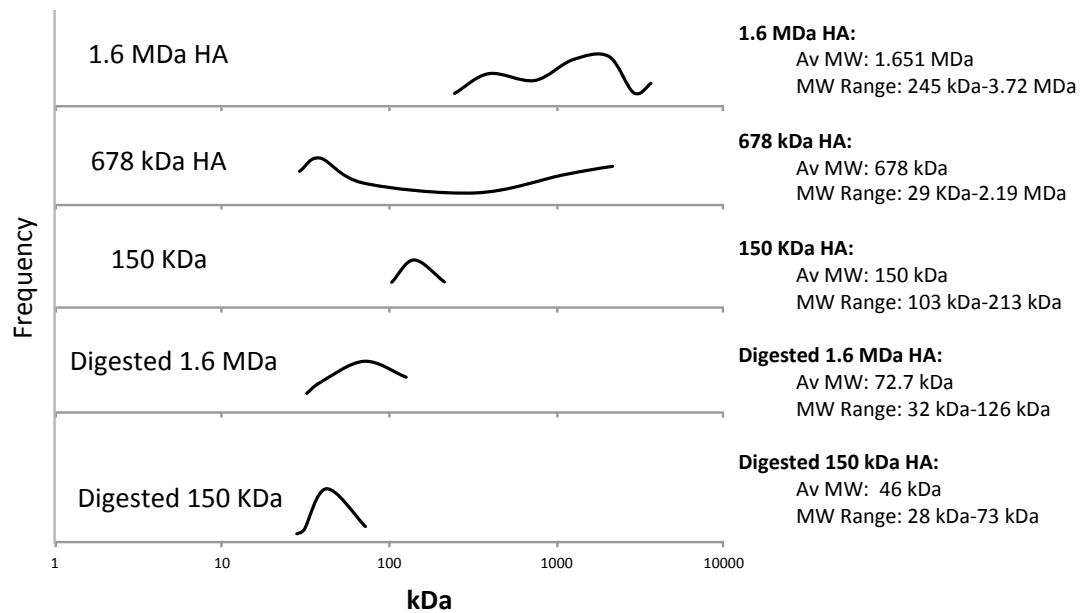

**Figure 1:** HA MW estimation using agarose gel electrophoresis.

Supplement: S1 Fig — (PDF) [file pone.0147868.s002.pdf]
