# Supplementary material for: A Systematic Study of the Effect of Different Molecular Weights of Hyaluronic Acid on Mesenchymal Stromal Cell-Mediated Immunomodulation
Source: PLoS One. 2016 Jan 28;11(1):e0147868. doi: 10.1371/journal.pone.0147868 (PMC4731468; doi:10.1371/journal.pone.0147868)
Supplement: S2 Fig — (PDF) [file pone.0147868.s003.pdf]

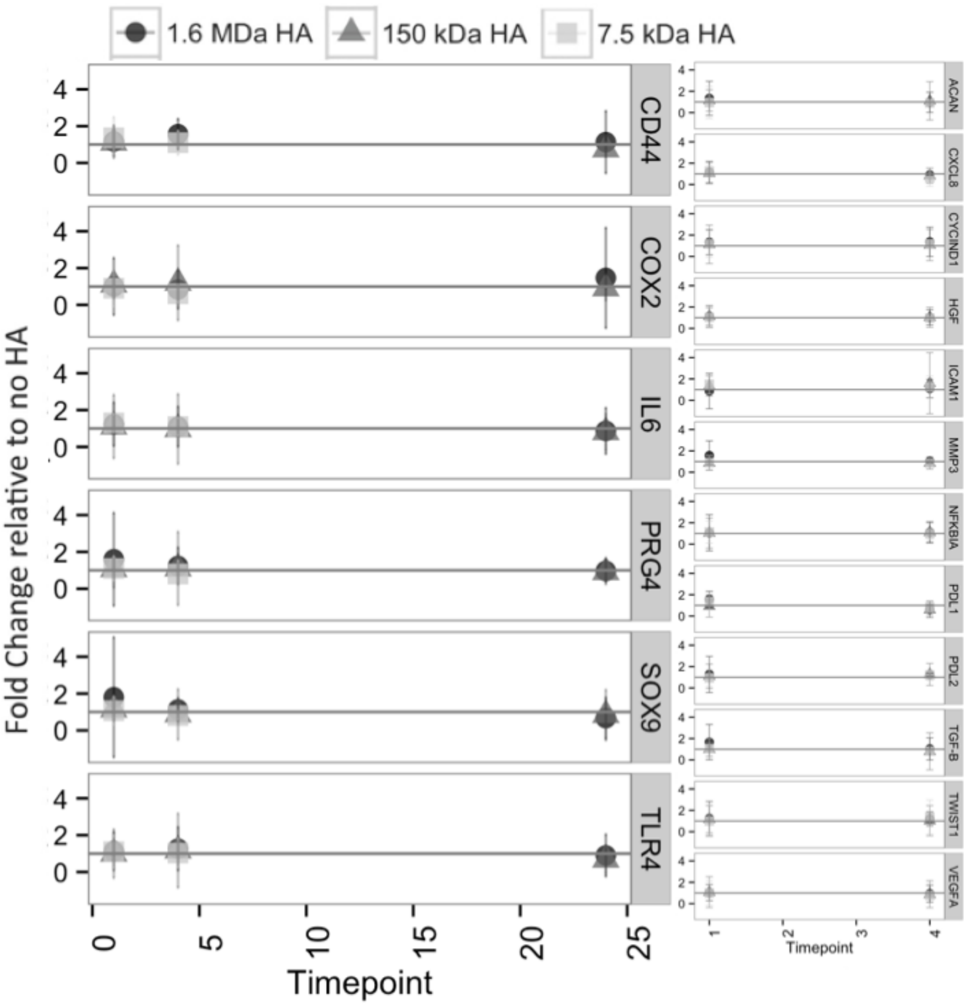

Figure 2: Gene expression in MSCs after 1,4 and 24 hours of exposure to different HAs. Line indicates basal transcript levels (fold change=1). Each dot indicates the average from MSCs from 3 different donors. Error bar indicates 95% CI.
