# Supplementary material for: A Systematic Study of the Effect of Different Molecular Weights of Hyaluronic Acid on Mesenchymal Stromal Cell-Mediated Immunomodulation
Source: PLoS One. 2016 Jan 28;11(1):e0147868. doi: 10.1371/journal.pone.0147868 (PMC4731468; doi:10.1371/journal.pone.0147868)
Supplement: S3 Fig — (PDF) [file pone.0147868.s004.pdf]

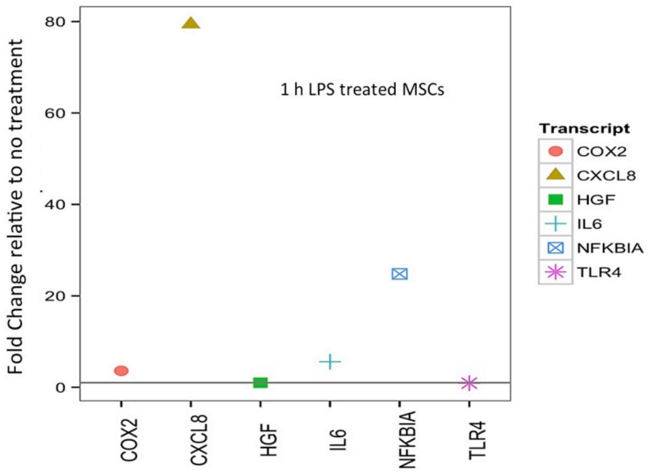

Figure 3: Gene expression in MSCs after 1 hour exposure to 100 ng/ml LPS. Line indicated basal transcript levels (fold change=1).
