# Supplementary material for: A Systematic Study of the Effect of Different Molecular Weights of Hyaluronic Acid on Mesenchymal Stromal Cell-Mediated Immunomodulation
Source: PLoS One. 2016 Jan 28;11(1):e0147868. doi: 10.1371/journal.pone.0147868 (PMC4731468; doi:10.1371/journal.pone.0147868)
Supplement: S4 Fig — (PDF) [file pone.0147868.s005.pdf]

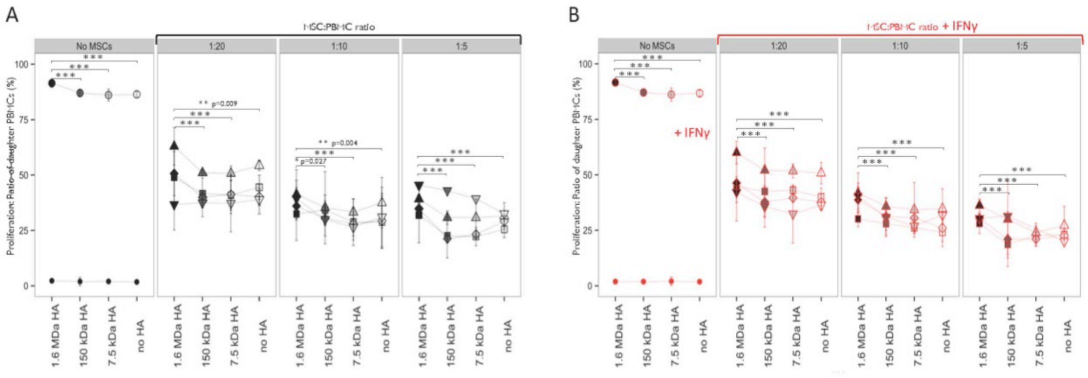


**Figure 4:** Effect of different MWs of HA on the MSC-mediated inhibition of PBL proliferation. (A) and (B) PBL proliferation from the whole PBMC population. (A) Normal conditions. (B) IFNγ supplemented conditions. • or • indicate resting PBLs; ○ Activated PBLs (PBMCs alone); □ MSC donor1, ◇ donor 2, ▽ donor 3 and △ donor 4. Shapes filled with black stand for 1.6 MDa HA; dark gray, 150 kDa HA; light gray, 7.5 kDa HA and white, no HA. Each shape/dot indicates the mean of three or four (in PBMCs alone groups) experimental replicates with error bars representing 95% CI. Statistical differences in the presence of MSCs always account for 4 MSC donors. * p<0.05, ** p<0.01 and ***<0.001.
