# Supplementary material for: A Systematic Study of the Effect of Different Molecular Weights of Hyaluronic Acid on Mesenchymal Stromal Cell-Mediated Immunomodulation
Source: PLoS One. 2016 Jan 28;11(1):e0147868. doi: 10.1371/journal.pone.0147868 (PMC4731468; doi:10.1371/journal.pone.0147868)
Supplement: S5 Fig — (PDF) [file pone.0147868.s006.pdf]

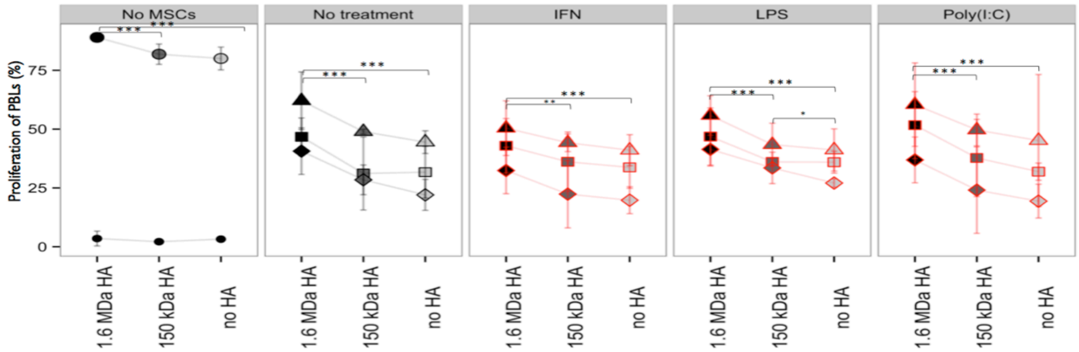

Figure 5: Effect of different MWs of HA on the MSC-mediated inhibition of PBL proliferation. Proliferation of PBLs from the whole PBMC population in normal conditions using non-pre-stimulated and IFN(γ), LPS and Poly(I:C) pre-stimulated MSCs. • **or** • indicate resting PBMCs; ○ Activated PBLs (PBMCs alone); □MSC donor1, ◇ donor 2, ▽ donor 3 and △ donor 4. Shapes filled with black stand for 1.6 MDa HA; dark gray, 150 kDa HA; light gray, 7.5 kDa HA and white, no HA. Each dot indicates the mean of three (in PBMCs alone groups) experimental replicates with error bars representing 95% CI. Statistical differences in the presence of MSCs account for 3-4 MSC donors. * p<0.05, ** p<0.01 and ***<0.001. MSCs were pre-stimulated, before adding PBMCs (MSC:PBMC ratio 1:10), with 500 U/ml IFNγ (24 hours), 100 ng/ml LPS (1 hour) or 1 µg/ml Poly(I:C) (24 hours), similar to Waterman et al[1].

1. Waterman RS, Tomchuck SL, Henkle SL, Betancourt AM. A new mesenchymal stem cell (MSC) paradigm: polarization into a pro-inflammatory MSC1 or an Immunosuppressive MSC2 phenotype. *PloS One*. 2010;5(4):e10088. doi:10.1371/journal.pone.0010088.
