# Supplementary material for: A Systematic Study of the Effect of Different Molecular Weights of Hyaluronic Acid on Mesenchymal Stromal Cell-Mediated Immunomodulation
Source: PLoS One. 2016 Jan 28;11(1):e0147868. doi: 10.1371/journal.pone.0147868 (PMC4731468; doi:10.1371/journal.pone.0147868)
Supplement: S6 Fig — (PDF) [file pone.0147868.s007.pdf]

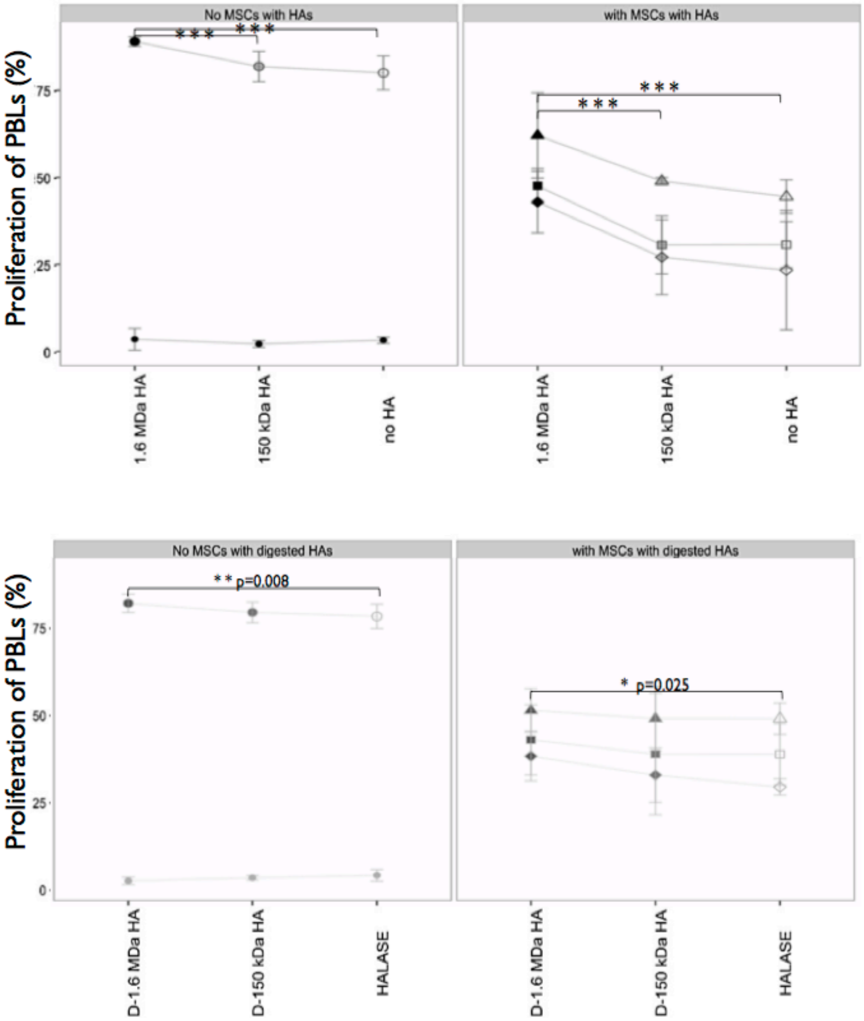

Figure 6: Proliferation of PBLs in medium containing HAs (Top) or Hyaluronidase-digested HAs (Bottom) with or without MSCs. • indicate resting PBMCs; ○ Activated PBLs (PBMCs alone); □MSC donor1, ◇ donor 2, and △ donor 3. Error bars indicate 95% CI. * p<0.05, **<0.01 and ***<0.001.
