# Supplementary material for: A Systematic Study of the Effect of Different Molecular Weights of Hyaluronic Acid on Mesenchymal Stromal Cell-Mediated Immunomodulation
Source: PLoS One. 2016 Jan 28;11(1):e0147868. doi: 10.1371/journal.pone.0147868 (PMC4731468; doi:10.1371/journal.pone.0147868)
Supplement: S7 Fig — (PDF) [file pone.0147868.s008.pdf]

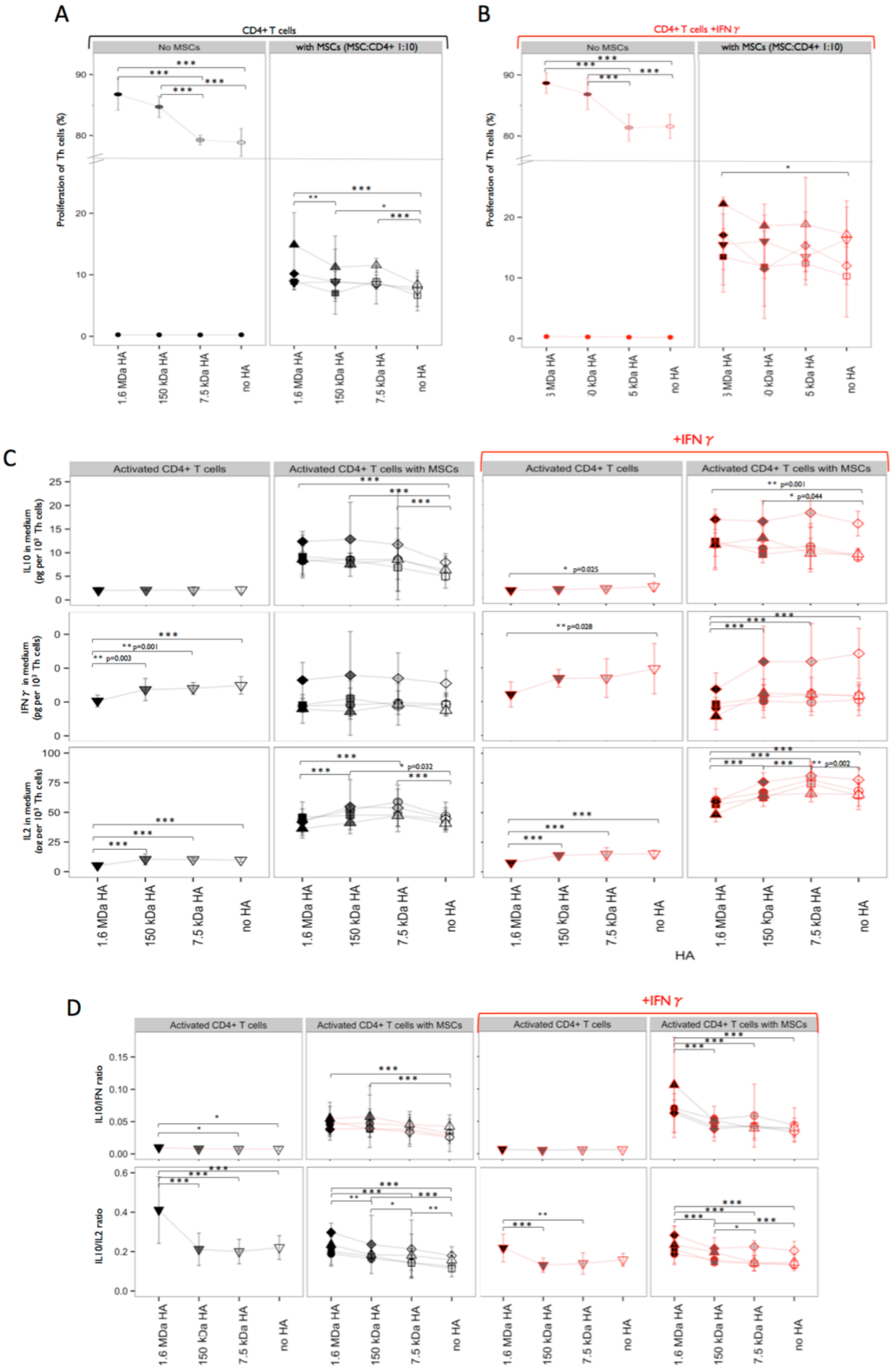

Figure 7: Effect of HAs on the interaction of MSCs and Th (CD4+ T cells) cells. (A) and (B) Proliferation measurements are shown as the level of daughter cells from the whole Th cell population. (E) IL10, IFNγ and IL2 levels in medium from Activated Th cells with or without MSCs and with or without IFNγ in medium. (F) Ratios of IL10/IFNγ, IL10/IL2 and IFNγ/IL2.○ Th cells alone, resting cells shown in dark small shape (•); □MSC sample 1, ◇sample 2, ▽sample 3 and △ sample4. Shapes filled with black stand for 1.6 MDa HA; dark gray, 150 kDa HA; light gray, 7.5 kDa HA and white, no HA. Each shape/dot indicates the mean of three or four (with no MSCs) experimental replicates with error bars representing 95% CI. * $p<0.05$, ** $p<0.01$ and ***<0.001.
