# Supplementary material for: A Systematic Study of the Effect of Different Molecular Weights of Hyaluronic Acid on Mesenchymal Stromal Cell-Mediated Immunomodulation
Source: PLoS One. 2016 Jan 28;11(1):e0147868. doi: 10.1371/journal.pone.0147868 (PMC4731468; doi:10.1371/journal.pone.0147868)
Supplement: S8 Fig — (PDF) [file pone.0147868.s009.pdf]

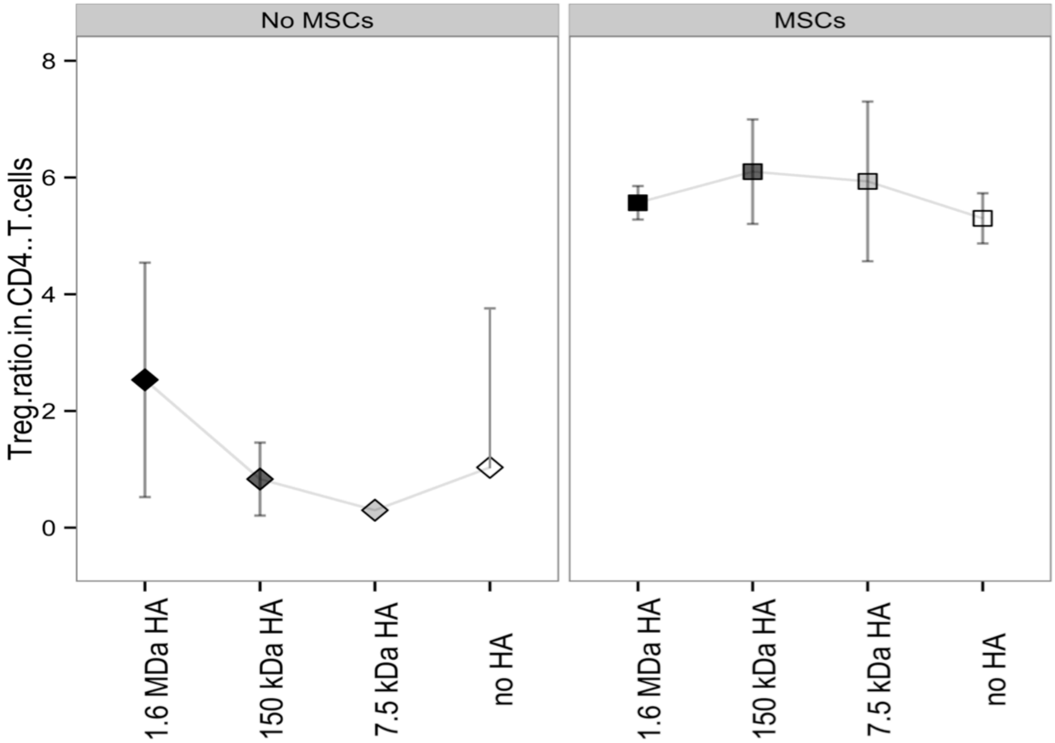

Figure 8: Effect of HAs on the MSC mediated induction of Tregs. Frequency of Tregs (CD25+CD127-) within CD4+ T cells is shown. ◇ CD4+ T cells alone; □ CD4+ T cells and MSCs. Shapes filled with black stand for 1.6 MDa HA; dark grays, digested-(D) 1.6 MDa HA or 150 kDa HA; light gray, 7.5 kDa HA and white, no HA. Each shape/dot indicates the mean of three experimental replicates with error bars representing 95% CI. * $p<0.05$, ** $p<0.01$ and ***<0.001.
