# Supplementary material for: A Systematic Study of the Effect of Different Molecular Weights of Hyaluronic Acid on Mesenchymal Stromal Cell-Mediated Immunomodulation
Source: PLoS One. 2016 Jan 28;11(1):e0147868. doi: 10.1371/journal.pone.0147868 (PMC4731468; doi:10.1371/journal.pone.0147868)
Supplement: S9 Fig — (PDF) [file pone.0147868.s010.pdf]

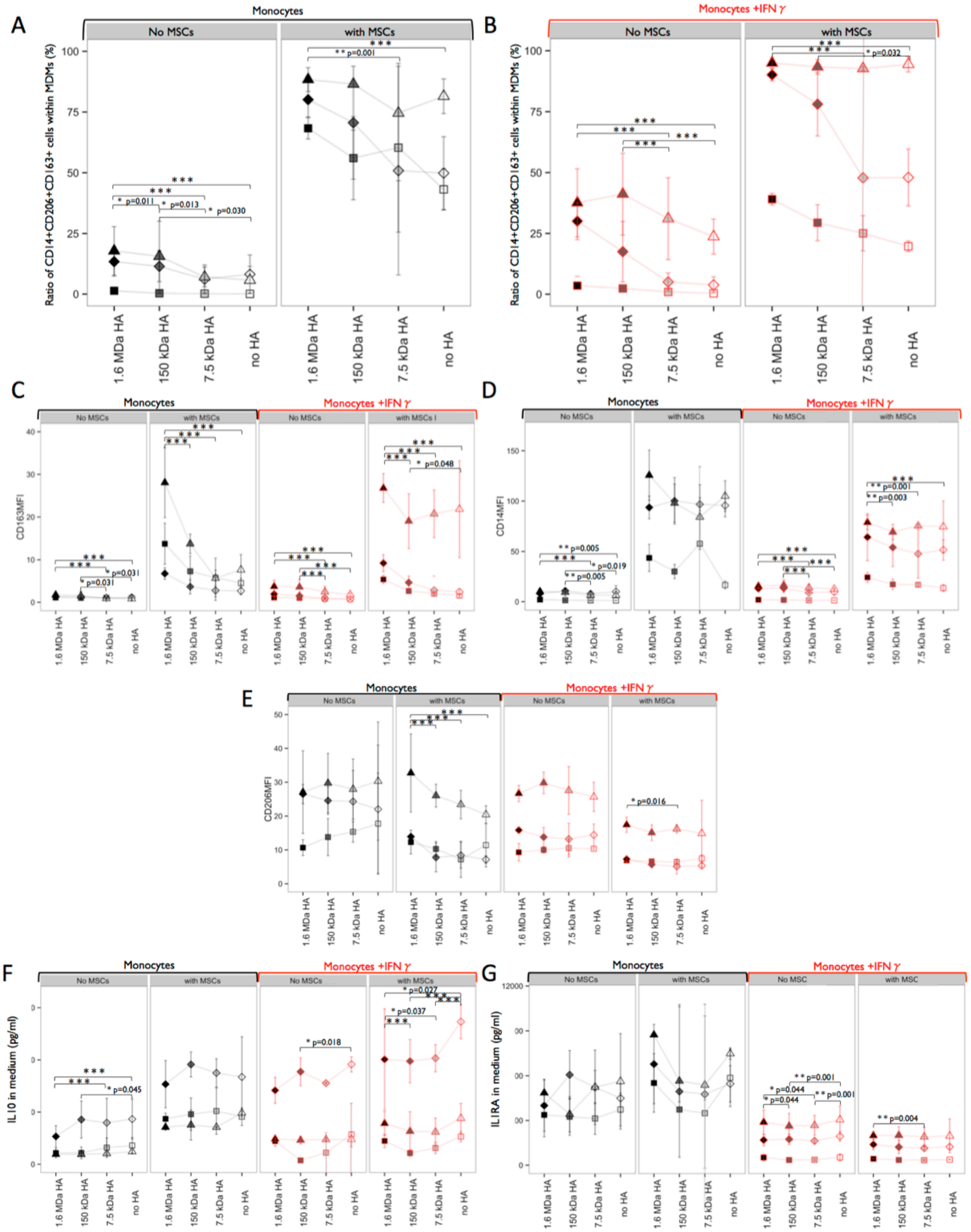

Figure 9: Effect of HAs on MSC-mediated induction of M2 MDMs. (A) and (B) Frequency of M2 MDMs within the whole MDM population. (C), (D) and (E), Mean fluorescence intensity (MFI) levels of CD163, CD14 and CD206 from whole MDM population. (F) and (G) Levels of secreted IL10 and IL1RA as measured by ELISA. □ Experiment 1;◇ Experiment 2; and △ Experiment 3; each experiment had a different MSC and monocyte donor. Shapes filled with black stand for 1.6 MDa HA; dark gray 150 kDa HA; light gray, 7.5 kDa HA and white, no HA. Each shape/dot indicates the mean of three experimental replicates with error bars representing standard deviation. * p<0.05, ** p<0.01 and ***<0.001.
