# Supplementary material for: A Systematic Study of the Effect of Different Molecular Weights of Hyaluronic Acid on Mesenchymal Stromal Cell-Mediated Immunomodulation
Source: PLoS One. 2016 Jan 28;11(1):e0147868. doi: 10.1371/journal.pone.0147868 (PMC4731468; doi:10.1371/journal.pone.0147868)
Supplement: S1 Table — (PDF) [file pone.0147868.s011.pdf]

Table 1: Primer nucleotide sequences of the tested transcripts

Function	Transcript	Forward	Reverse
HA receptor	**CD44**	CGT GGA ATA CAC CTG CAA AG	CGG ACA CCA TGG ACA AGT TT
Trophic	**IL6**	TCT CCA CAA GCG CCT TCG	CTC AGG GCT GAG ATG CCG
Anti-inflammatory	**COX2**	ATA AGC GAG GGC CAG CTT TC	CGC AGT TTA CGC TGT CTA GC
Chondrogeneic	**SOX9**	ATG CAA GCA TGT GTC ATC CA	AGG TCT GTC AGT GGG CTG AT
Joint lubricant	**PRG4**	AGG CCC CAT GTG TTC ATG C	GCG CAA AGT AGT CAG TCC ATC T
HA fragments receptor?	**TLR4**	TGA GCA GTC GTG CTG GTA	CAG GGC TTT TCT GAG TCG
HA receptor	**ICAM1**	AGG GTA AGG TTC TTG CCC AC	TGA TGG GCA GTC AAC AGC TA
Proliferation	**CYCLIND1**	ACC TGG ATG CTG GAG GTC T	GCT CCA TTT GCA GCA GCT C
Pro-inflammatory	**NFKBIA**	TCA TGG ATG ATG GCC AAG T	GTC AAG GAG CTG CAG GAG AT
Angiogeneic, chemotactic	**CXCL8**	AAA TTT GGG GTG GAA AGG TT	TCC TGA TTT CTG CAG CTC TGT
Chondrolysis	**MMP3**	CAA AGC TTC AGT GTT GGC TG	GGC CAG GGA TTA ATG GAG AT
Chondrogenic	**ACAN**	ACT CTG GGT TTT CGT GAC TCT	ACA CTC AGC GAG TTG TCA TGG
Angiogeneic, chondrolysis	**VEGFA**	CTA CCT CCA CCA TGC CAA GT	AGC TGC GCT GAT AGA CAT CC
Anti-inflammatory	**PDL1**	TCA ATG CCC CAT ACA ACA A	TGC TTG TCC AGA TGA CTT CG
Anti-inflammatory	**PDL2**	GTA CAT AAT AGA GCA TGG CAG CA	CCA CCT TTT GCA AAC TGG CTG T
Anti-inflammatory, trophic	**HGF**	TGG TTT TAA TGA AGC TTG CCA G	GAG ATG TGC CAC TCG TAA TAG G
Anti-inflammatory Chondrogeneic	**TGFβ**	ACT GCG GAT CTC TGT GTC AT	AGT AGT GTT CCC CAC TGG TCC
Anti-inflammatory	**TSG6**	AGC ACG GTC TGG CAA ATA CA	ATC CAT CCA GCA GCA CAG AC
Anti-inflammatory	**IDO**	GCC CTT CAA GTG TTT CAC CAA	CCA GCC AGA CAA ATA TAT GCG A
Chondrogeneic	**COL2A1**	GTC CTC TGC GAC GAC ATA ATC	CCT TGA TGT CTC CAG GTT CTC C
Anti-inflammatory	**IL1RA**	AGC ATG AGG CTC AAT GGG TA	AAA TCC AGC AAG ATG CAA GC
Anti-inflammatory	**IL10**	CGAGATGCCTTCAGCAGAGT	CGCCTTGATGTCTGGGTCTT
Pro-inflammatory	**TNF**	GCC AGA GGG CTG ATT AGA GA	TCA GCC TCT TCT CCT TCC TG
Pro-inflammatory	**CXCL13**	CAA TGA AGC GTC TAG GGA TAA AG	CAG TCC AAG GTG TTC TGG
Chondrolysis	**MMP13**	TCA CCA ATT CCT GGG AAG TCT	TCA GGA AAC CAG GTC TGG AG
Housekeeping Gene	**B2M**	CTC CGT GGC CTT AGC TGT G	TTT GGA GTA CGC TGG ATA GCC T

Interleukin 6, IL6; sex determining region Y-box 9, SOX9; Proteoglycan 4, PRG4; Toll-like receptor 4, TLR4; Intercellular adhesion molecule 1, ICAM1; Twist-related protein 1, TWIST1; nuclear factor of kappa light polypeptide gene enhancer in B-cells inhibitor, alpha, NFKBIA; Interleukin 8, CXCL8; matrix metalloproteinase-3 and 1, MMP-3 and -13; Aggrecan, ACAN; Vascular endothelial growth factor alpha, VEGFA; Programmed death-ligand 1 and 2, PDL1 and PDL2; Hepatocyte growth factor, HGF; Transforming growth factor beta, TGFβ; Tumor necrosis factor-inducible gene 6 protein, TSG6; indoleamine 2,3-dioxygenase, IDO; Collagen type II alpha 1, COL2A1; Tumour necrosis factor alpha; TNF; chemokine ligand 13, CXCL13.
