# Supplementary material for: A Systematic Study of the Effect of Different Molecular Weights of Hyaluronic Acid on Mesenchymal Stromal Cell-Mediated Immunomodulation
Source: PLoS One. 2016 Jan 28;11(1):e0147868. doi: 10.1371/journal.pone.0147868 (PMC4731468; doi:10.1371/journal.pone.0147868)
Supplement: S2 Table — (PDF) [file pone.0147868.s012.pdf]

Abbreviations used in this paper	
ACAN	Aggrecan
B2M	Beta-2-Microglobulin
cDNA	Complementary DNA
COL2A1	Collagen type II Alpha 1
COX2	Cyclooxygenase 2
CXCL13	Chemokine ligand 13
CXCL8	Neutrophil chemotactic factor or Interleukin 8
CYCLIND1	Twist-related protein 1
DMEM	Dulbecco's Modified Eagle Medium
FBS	Fetal Bovine Serum
GM-CSF	Granulocyte-Macrophage Colony-Stimulating Factor
HA	Hyaluronic Acid; Hyaluronan
HGF	Hepatocyte Growth Factor
hHA	High Molecular Weight HA: 1.6 MDa HA
ICAM1	Intercellular adhesion molecule 1
IDO	Indoleamine 2,3-DiOxygenase
IFNγ	Interferon γ
IL	Interleukin
IL1RA	Interleukin 1 Receptor Antagonist
LPS	Lipopolysaccharide
M-CSF	Macrophage Colony-Stimulating Factor
MDM	Monocyte-Derived Macrophage
MMP	Matrix Metalloproteinase
MSC	Mesenchymal Stromal Cell
MW	Molecular Weight
NFKBIA	Nuclear Factor of Kappa light polypeptide gene enhancer in B-cells Inhibitor Alpha
OA	Osteoarthritis; Osteoarthritic
PBL	Peripheral Blood Lymphocytes
PBMC	Peripheral Blood Mononuclear Cells
PCR	Polymerase Chain Reaction
PDL	Programmed Death-Ligand
PRG4	Proteoglycan 4
RNA	Ribonucleic acid
RPMI	Roswell Park Memorial Institute
RT	Reverse Transcription
SF	Synovial Fluid
SOX9	Sex determining region Y-box 9
TGFβ	Transforming Growth Factor β
Th	T helper
TLR	Toll-Like Receptor
TNF	Tumour necrosis factor alpha
Tregs	T regulatory cells
TSG6	Tumor necrosis factor-inducible Gene 6 protein
VEGFA	Vascular endothelial growth factor alpha
